# A decade on: where is the UK poultry industry for emergency on-farm killing?

**DOI:** 10.1016/j.psj.2023.102604

**Published:** 2023-02-21

**Authors:** Jasmine M. Clarkson, Alexandra Paraskevopoulou, Jessica E. Martin

**Affiliations:** ⁎ School of Biodiversity, One Health and Veterinary Medicine, College of Medical Veterinary and Life Sciences, University of Glasgow, Glasgow, United Kingdom; † School for Natural and Environmental Sciences, Newcastle University, Newcastle upon Tyne, United Kingdom; ‡ The Royal (Dick) School of Veterinary Studies and The Roslin Institute, The University of Edinburgh, Edinburgh, United Kingdom

**Keywords:** chicken, turkey, euthanasia, cervical dislocation, animal welfare

## Abstract

Millions of poultry are farmed intensively every year across the United Kingdom (UK) to produce both meat and eggs. There are inevitable situations that require birds to be emergency killed on farm to alleviate pain and suffering. In Europe and the UK, emergency methods are regulated by the European Council Regulation (EC) No. 1099/2009 and The Welfare of Animals at the Time of Killing Regulations (England 2015; Scotland 2012; Wales and Northern Ireland 2014). Cervical dislocation has been reported to be the most widely used method prior to these legislative changes which took place from 1 January 2013. Based on limited scientific evidence and concern for bird welfare, these legislative changes incorporated restrictions based on bird weight for both manual (≤3 kg) and mechanical (≤5 kg) cervical dislocation, and introduced an upper limit in the number of applications for manual cervical dislocation (up to 70 birds per person per day). Furthermore, it removed methods which showed evidence of crushing injury to the neck. However, since legal reform new scientific evidence surrounding the welfare consequences of cervical dislocation and the development of novel methods for killing poultry in small numbers on farm have become available. Whether the UK poultry industry have adopted these novel methods, and whether legislative reform resulted in a change in the use of cervical dislocation in the UK remains unknown. Responses from 215 respondents working across the UK poultry industry were obtained. Despite legal reform, manual cervical dislocation remains the most prevalent method used across the UK for killing poultry on farm (used by 100% of farms) and remains the preferred method amongst respondents (81.9%). The use of alternative methods such as Livetec Nex® and captive bolt guns were available to less than half of individuals and were not frequently employed for broilers and laying hens. Our data suggests there is a lack of a clear alternative to manual cervical dislocation for individuals working with larger species and a lack of gold standard methodology. This risks bird welfare at killing and contributes to inconsistency across the industry. We suggest providing stakeholders with practical alternatives prior to imposing legislative changes and effective knowledge transfer between the scientific community and stakeholders to promote positive change and protect bird welfare.

## INTRODUCTION

Poultry remain the predominant land species slaughtered every year for meat production in addition to their use for egg production. In the United Kingdom (UK) alone, approximately 100 million meat birds are slaughtered each month (∼25 million every week) and there were 39 million laying hens in 2021 (Department for Environment Food & Rural Affairs Defra 2021). Consequently, to meet consumer demand and high yield, 95% of broilers are reared under intensive farming systems which have the capacity to house more than 40,000 birds (Dal Bosco et al., 2021). As such, there are inevitable situations that require birds to be killed on-farm to alleviate pain and suffering of an individual, for disease management practices or for production management purposes. However, methods used for emergency killing on farm pose concern for animal welfare due to the capacity for birds to experience pain and distress during handling and prior to loss of consciousness, meaning a number of on-farm killing methods have undergone detailed welfare assessment in recent years (Bader et al., 2014; Martin et al., 2016,2018a,b, 2019; Woolcott et al., 2018a; Hernandez et al., 2019a; Jacobs et al., 2019; Boyal et al., 2020). (e.g., Erasmus et al., 2010a,b; Bader et al., 2014; Martin et al., 2016, 2018a, 2019; Hernandez et al., 2019b; Jacobs et al., 2019)

According to the European Council Regulation (EC) No. 1099/2009 of 24 September 2009 on the protection of animals at the time of killing (PATOK) (EC, 2009) and the UK national regulations that enforce PATOK, The Welfare of Animals at the Time of Killing Regulations (England 2015; Scotland 2012; Wales and Northern Ireland 2014 (WATOK) (UK Government, 2015)), the permitted emergency methods for killing poultry allow the use of cervical dislocation, percussive devices, decapitation, overdose of a lethal drug, electrical water bath stunning and the use of gases. However, previous reports have suggested that the primary method for killing poultry is cervical dislocation (Sparrey et al., 2014; Martin, 2015; Watteyn et al., 2020) due to its apparent ease of application and practicality (Erasmus et al., 2010a; Martin et al., 2016, 2018b). Cervical dislocation (i.e., neck dislocation) can be split into 2 main categories: mechanical and manual; based on the aid of equipment (mechanical) or not (manual) (Bader et al., 2014; Martin et al., 2016, 2019; EFSA et al., 2019; Boyal et al., 2020). Mechanical cervical dislocation involves the use of a tool to aid in the dislocation of the neck for example, using a broomstick (Sparrey et al., 2014) or recently available Livetec Nex® (Livetec Systems ltd., Bedford, Bedfordshire, United Kingdom) (Livetec Systems ltd., 2018; Martin et al., 2019). In the case of manual cervical dislocation, the operator uses their hands rather than any dedicated equipment to dislocate the neck of the bird. This involves grasping the legs of the bird in one hand and stretching the neck by pulling on the head while applying a backward rotational force to the skull (Sparrey et al., 2014), however variations in technique have been documented (Martin et al., 2018a). Whether manual or mechanical cervical dislocation, both methods when applied correctly, are designed to cause death by cerebral ischemia and extensive damage to the spinal cord (Bader et al., 2014; Martin et al., 2016,^2019^), with the success of application highly dependent on location of the dislocation and whether one or both carotid arteries are severed (Martin et al., 2019,^2021^).

Cervical dislocation has been reported as the traditional and most common method for emergency killing poultry on farm (Sparrey et al., 2014; Martin, 2015), especially for the primary poultry species, chickens (layer hens and broilers). Prior to legislative changes, there was growing evidence that cervical dislocation was unlikely to result in immediate loss of consciousness, highlighting concerns in relation to potential welfare impacts (e.g., Gregory and Wotton, 1990; Erasmus et al., 2010b). These initial findings were used as evidence to support changes in legislation (EC 1099/2009), which became enforced as of 1 January, 2013, which attempted to restrict the use of cervical dislocation methods. The restrictions involved 2 approaches: live bird weight thresholds for manual (≤3 kg birds only) and mechanical (≤5 kg birds) cervical dislocation, as well as an upper limit of applications for manual cervical dislocation (up to 70 birds per person per day). These live weight and number limits appear to have no justification from the scientific literature prior to the legislation drafting or enforcement. In reality, both approaches to restrict the use of cervical dislocation may have had little impact on the poultry industry in the UK and EU, as the daily number limit for applications is unlikely to be reached (e.g., daily mortality 0.02–0.6% in broilers (Xin et al., 1994; Tabler and Berry, 2001)) and given layer hens and broilers are the primary poultry bird groups, the live weight limit of 3 kg is unlikely to be exceeded (Anene et al., 2020; Department for Environment Food & Rural Affairs Defra, 2021).

The principal concern with regards to manual cervical dislocation arises due to questions surrounding the efficacy of the method and time to loss of consciousness, which has remained the focus of ongoing debate across the scientific community (Gregory and Wotton, 1990; Erasmus et al., 2010a,b; Bader et al., 2014; Martin et al., 2016,2018a,^2021^; Bandara et al., 2019a; Jacobs et al., 2019; Baker-Cook et al., 2021a; Stiewert et al., 2021). This is further exacerbated by grouping together all cervical dislocation methods (manual and mechanical) in scientific studies and generalizing their findings. For example, there are very few published studies exploring the welfare impacts of genuine manual cervical dislocation, including the analysis of electrical activity of the brain via electroencephalography and/or behavioral reflex data (Erasmus et al., 2010a; Martin et al., 2016,^2019^; Woolcott et al., 2018a; Hernandez et al., 2019a,b; Jacobs et al., 2019) to ascertain time to loss of consciousness and behavioral indicators of pain and suffering. The majority of studies exploring cervical dislocation killing techniques in poultry relied upon applying a mechanical version instead (e.g., killing cone or Burdizzo) (Gregory and Wotton, 1990; Erasmus et al., 2010a,b). The reason for this is not clear, but an attempt to standardize method application for experimental rigor would be feasible. Critically, any studies evaluating true manual cervical dislocation occurred following the EC 1099/2009 drafting and enforcement.

Whether or not the legislation change did restrict the use of cervical dislocation, the scenario is further complicated by the availability of alternatives for emergency killing. In 2009, the Farm Animal Welfare Council (now Animal Welfare Committee) recommended research to explore current and novel methods for killing poultry in small numbers on farm (Farm Animal Welfare Council (FAWC), 2009). Since then, a number of devices have been developed and become commercially available (e.g., CASH Small Animal Tool® [Frontmatec Accles & Shelvoke, Sutton Coldfield, UK], Turkey Euthanasia Device, Livetec Nex®, Koechner Euthanasia Device, Zephyr E and Zephyr EXL (Livetec Systems ltd., 2018; Martin et al., 2018b,^2019^; Woolcott et al., 2018b; Hernandez et al., 2019a; Boyal et al., 2020; Stiewert et al., 2021; Frontmatec accles & Shelvoke, 2022)); however, whether the poultry industry in the UK and across the EU have adopted these methods and to what scale is relatively unknown. To our knowledge, only 2 studies have surveyed the killing methods used for poultry on-farm: 1) a pilot study in the UK in 2011, prior to the EC 1099/2009 enforcement (Martin, 2015) and 2) in Belgium in 2017, post regulation enforcement (Watteyn et al., 2020). Therefore, little remains known outside of the poultry industry regarding method availability, use and reasons behind method selection, and specifically how regulation reform impacted practices across the UK and Europe. The Flemish study demonstrated manual cervical dislocation remained the most common method employed for killing broiler chickens and turkeys despite the introduction of legal restrictions between 2011 and 2017 (Watteyn et al., 2020). However, as the authors highlighted this work was limited by a small sample size (a total of 44 participants) due to low response rates of uptake across poultry veterinarians and producers across Belgium at the time. The popularity of manual cervical dislocation remains noticeable and is consistent with observations in the UK from 2011 (Martin, 2015), perhaps suggesting that the legislation change did not result in the intended widespread changes to on-farm killing methods for poultry. The reasons for method selection and preference remain unknown but could be attributed to several reasons including factors such as perceived humaneness, legality, ease of application, level of effectiveness and safety of the stock-workers. Therefore, the purpose of this survey was to fill the gap in knowledge and establish the current situation in the UK for the first time since the legislative reform (EU 1099/2009) in 2013. We aimed to determine the availability and current use of killing methods for killing commercial poultry on farm in the UK and identify reasons behind these choices.

## MATERIALS AND METHODS

### Ethical Approval

The project was approved by the University of Edinburgh Human Ethical Review Committee (reference: HERC_714-21), in accordance with relevant guidelines and regulations (General Data Protection Regulation (GDPR) (EU 2016/679), 2018).

### Recruitment and Procedure

A short anonymous survey was designed and distributed electronically among members of the UK poultry industry using the Jisc Online Survey tool. To be eligible to take part in the study, participants had to be at least 18 yr of age, reside in the UK and work with poultry. The survey was disseminated through 2 main routes: 1) a URL link to the online survey was distributed with an invitation email to established email contacts (where consent was provided) within the commercial UK poultry industry, industry partners and representative bodies (British Poultry Council, British Egg Industry Council, World Poultry Science Association (UK branch)); 2) sharing of the URL link on social networking sites via professional academic accounts (e.g., Facebook, Twitter, LinkedIn). Both routes encouraged further distribution of the URL link to others who may be interested in participating. The survey was open from May 2021 to August 2021. Interested participants were initially provided with a brief background to the research including information on the structure and content of the questionnaire, data protection, anonymity and consent details on the welcoming page of the URL link. Those who did not provide consent to participate were exited here.

### Survey Design

The survey comprised a total of 25 questions divided into 3 sections: 1) general demographics; 2) identification and preference of killing methods used and available; and 3) attitudes towards the relevant legislation in the UK. See S1 in supplementary material for full survey. In the first section of the survey, the demographic multiple-choice questions gathered relevant information from respondents regarding their gender, age, country of residence, level of education and questions related to their work experience. For most questions throughout the survey, a “Prefer not to say” response option was included (Kulas and Stachowski, 2013).

The second section comprised of both multiple-choice, 6-point Likert scales for agreement and 10-point ordinal scales. To avoid the choice of a neutral option which increases social desirability and central tendency bias, the Likert scales were even numbered excluding the ‘neither agree nor disagree’ option (Nadler et al., 2015). In this section, responders were asked about their currently used, available and preferred killing methods. The responders were first asked general questions about the farming system and stage of poultry production they work in, the number of times they inspect birds in their care and how often they have to kill a bird. They were then asked the reasons required for a bird to be killed on-farm, which killing methods were permitted and available for use at their site and what their preferred killing methods were from predetermined multiple-choice selection lists, with an “other” category included. For example, to answer the questions regarding the killing methods (regardless of availability), 13 methods were listed (manual cervical dislocation, mechanical cervical dislocation methods (including: pliers, broomstick, cone and Livetec Nex®), blow to the head (blunt force trauma), overdose of anesthetic, captive bolt (cartridge and noncartridge), decapitation, electrical stun to kill, gas, and other). For each method, the responders were asked how often they use that method (with answer options: always, often, sometimes, rarely, never, or unknown). Another question focused on which method the respondents prefer (a single choice from the list of the 13 methods). In addition, responders were asked to rank a list of killing method properties based on the level of importance (rank 1 being of no importance and rank 10 being most important). For 4 specific killing methods (manual cervical dislocation, captive bolt (cartridge and noncartridge powered), mechanical cervical dislocation (Livetex Nex) and overdose of anesthetic), participants were asked to indicate to what extent they agree or disagree with statements for each killing method on a 6-point likert scale (with answer options: strongly disagree, moderately disagree, slightly disagree, slightly agree, moderately agree, strongly agree or unknown). These 4 killing methods were selected to include the predicted 3 most common killing methods used (based on results from the surveys in 2011 and 2017 (Martin, 2015; Watteyn et al., 2020)) and a perceived “gold-standard” killing method (overdose of anesthetic) which is the primary method undertaken by veterinary professionals across multiple species (Hernandez et al., 2019a).

The last section focused on the attitudes towards the current legislation in the UK and also comprised of 6-point Likert scales to ascertain agreement with prepared statements. In this section, the responders were asked to indicate to what extent they agree or disagree with each of 10 listed statements, regarding the weight and bird number restrictions imposed by the legislation changes in 2013 on manual and mechanical cervical dislocation.

### Statistical Analyses and Data Processing

Data was exported from Jisc Online Survey tool (Jisc, UK) as an Excel file format. All statistical analyses were conducted in R and R Studio (version 1.3.109331). Only responses from individuals who consented to and completed the full survey were included in the analysis. Responses were fully anonymous in accordance with the The Data Protection Act 2018 is the UK's implementation of the General Data Protection Regulation. All data was collated and processed within R using the tidyverse package (Wickham et al., 2019). All graphical summaries were created using ggplot2 (Wickham, 2016). Ranked data was analyzed using Cumulative Link Models using package ordinal and RVAideMemoire to compare mean ranks with the threshold set to equidistant (Christensen, 2019). Exploration of the influence of demographic factors was performed via models including fixed factors such as primary species (6 levels), sector (4 levels), and farming system (5 levels). We grouped primary bird species into 3 bird sizes based on slaughter/end of lay weights (small, medium, and large) and was included as a fixed factor in statistical modelling. Small birds were considered ≤3 kg (broilers and layer hens), medium birds (<3 to ≤5 kg) included broiler breeders, ducks and mixed species and large birds included turkeys (≥5 kg). Therefore, these bird sizes reflect upper weight limits, and it is possible that birds are killed before reaching these thresholds. Statistical significance was based on *P* < 0.05 threshold on the Χ_2_ statistical test. Pairwise comparisons were reported using estimated marginal means via the emmeans package (Lenth, 2021), with P values adjusted for multiplicity using the Tukey method where nonsignificant results are not reported. Reported values in the manuscript reflect estimated marginal means of ordinal data from emmeans package.

## RESULTS

### Participant Demographics

A total of 215 participants (Male: 89.3%, n = 192, Female: 10.7%, n = 23) across a diverse range of age categories (18–24 years old 8.4%, n = 18, 25–34 years old 40.5%, n = 87, 35–44 years old 17.2% n = 37, 45–54 years old 15.8%, n = 34, 55–64 years old 9.3%, n = 20, 65+ years old 8.8%, n = 19) completed the survey in full. Most responses came from participants residing in England (69.3%, n = 149), however we also obtained responses from other countries within the UK, including Scotland (16.3%, n = 35), Wales (10.2%, n = 22) and Northern Ireland (4.2%, n = 9). Overall, most responses came from producers (81.4%, n = 175) but responses from other sectors were obtained, including veterinary services (11.2%, n = 24), "other" (6.5%, n = 14 including nutritional and research, but other sector information were not always provided) and breeding (0.9%, n = 2). We found a diverse and relatively balanced range in experience across participants (2–5 yr 20.9%, n = 54, 6–11 yr 27.9%, n = 60, 12–17 yr 25.1%, n = 54, 18+ yr 26%, n = 56) with the majority educated to school level (GCSE or A level equivalent 61.4%, n = 132). However, some participants did report higher level qualifications including holding an undergraduate degree (29.3%, n = 63), postgraduate degree (8.4%, n = 18) or "other" (0.9%, n = 2). Most participants reported working primarily with broilers (56.7%, n = 122) however participants also worked with layer hens (18.6%, n = 40), turkeys (13%, n = 28), broiler breeders (6%, n = 13), mixed species (4.2%, n = 9), and ducks (1.4%, n = 3). We also obtained responses from different farming systems across all bird types, including indoor (noncaged) farms (76.3%, n = 164), free range (18.6%, n = 40), organic (2.3%, n = 5), mixed/other (2.3%, n = 5) and indoor caged (0.5%, n = 1) systems.

### Availability, Application, and Use of on Farm Dispatching Methods

Manual cervical dislocation was available to all respondents (100%, n = 215). In comparison, mechanical cervical dislocation was available to roughly half of participants (50.2%, n = 108) either through the provision of Livetec Nex® (42.8%, n = 92), a broomstick (4.2%, n = 9), pliers (2.8%, n = 6), or cone (0.5%, n = 1). Other methods available included captive bolt (26%, n = 56), blunt force trauma (1.9%, n = 4), overdose of anesthetic (8.4%, n = 18), decapitation (3.3%, n = 7), electrical stun to kill (1.9%, n = 4) and exposure to gas (2.3%, n = 5). However, method availability was influenced by bird size (Table 1). Individuals working with medium to large birds (>3 kg) stated greater availability to mechanical cervical dislocation and captive bolt compared to those working with small birds (≤3 kg) (Table 1).

**Table 1:** Number of respondents who reported killing method availability on their current poultry farm (and percentage availability (%) of those working with that bird size), according to bird size at slaughter/end of production (small; broilers and laying hens, medium; broiler breeders, ducks and mixed, large; turkeys).

Killing method	Small (<3 kg) (n = 162)	Medium (3–5 kg) (n = 25)	Large (>5 kg) (n = 28)
Mechanical cervical dislocation (all methods collated)	61 (37.7%)	23 (92.0%)	24 (85.7%)
Captive bolt	9 (5.6%)	19 (76.0%)	28 (100.0%)
Blunt force trauma	3 (1.9%)	1 (4.0%)	0 (0.0%)
Overdose of anesthetic	11 (6.8%)	7 (28.0%)	0 (0.0%)
Decapitation	7 (4.3%)	0 (0.0%)	0 (0.0%)
Electrical Stun-to-kill	4 (2.5%)	0 (0.0%)	0 (0.0%)
Gas	5 (3.1%)	0 (0.0%)	0 (0.0%)

Mechanical cervical dislocation represents the sum of the following devices (broomstick, pliers, cone, Nex) and captive bolt included both cartridge and noncartridge powered. Manual cervical dislocation availability is not included as it was available to all respondents (100%, n = 215).

**Figure 1:**
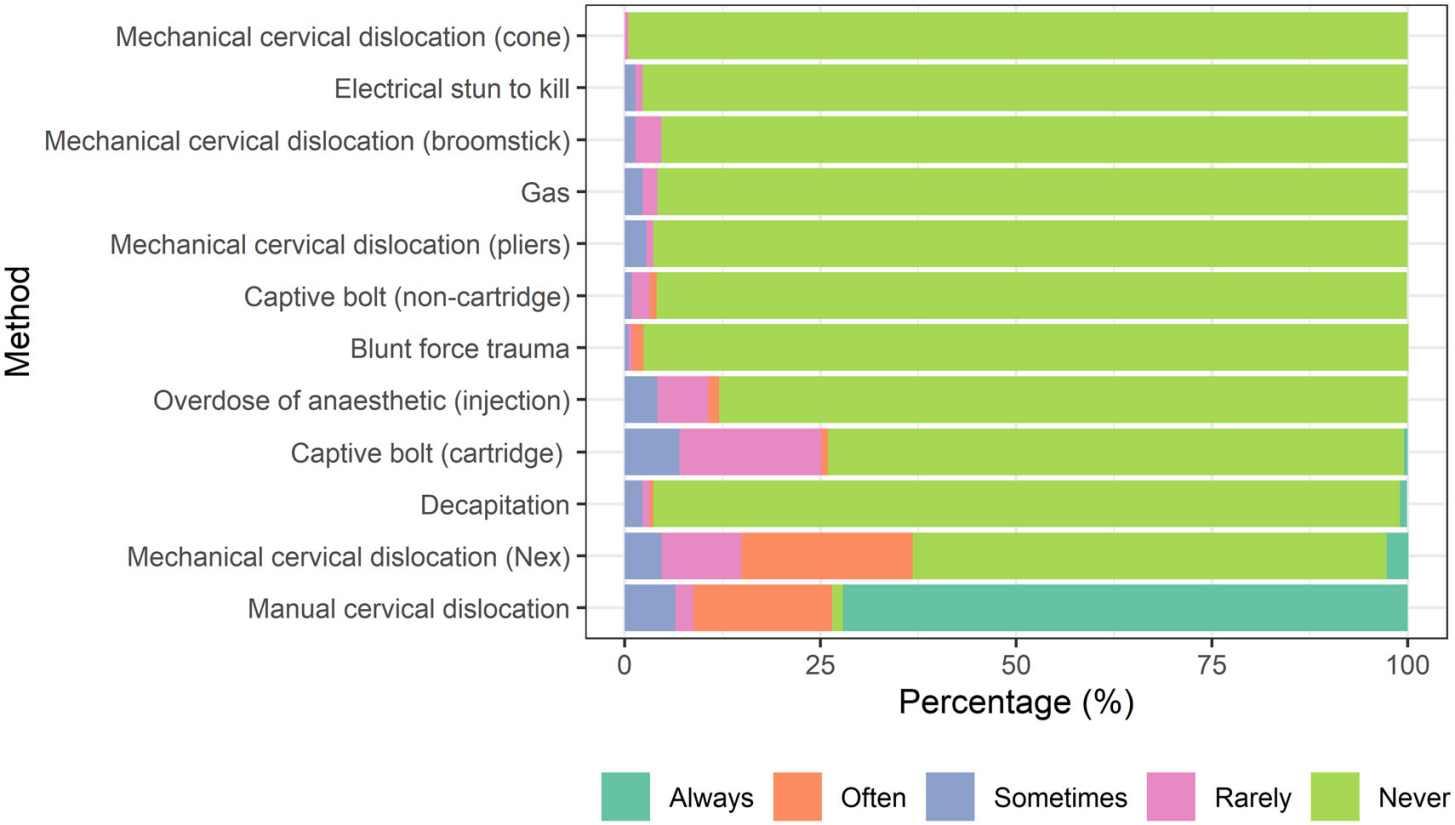
Mean percentage of participants stating frequency of use for a range of on-farm killing methods irrespective of bird weight. Responses range from always, often, sometimes, rarely, or never.

Most respondents reported having to kill an individual bird on farm daily (66.5%, n = 143), with the remainder reporting a couple of times a week (21.9%, n = 47), a couple of times a year (4.7%, n = 10), once a month (4.2%, n = 9), once a week (1.9%, n = 4) or never (0.9%, n = 2). In terms of application frequency, the most widely used killing method was manual cervical dislocation, whereby 72.1% (n = 155) of participants stated “always” using it (Figure 1). However, other methods were always selected by some participants including Livetec Nex® (2.8%, n = 6), captive bolt (cartridge) (0.5%, n = 1) or decapitation (0.9%, n = 2) (Figure 1).

However, the frequency of method application was highly dependent upon the weight of the species that individuals primarily reported working with (Table 2). We found more participants stating they always use manual cervical dislocation when working with small birds (≤3 kg) compared to medium birds (>3 to ≤5 kg) and large birds (≥5 kg) (Table 2). Instead, individuals working with medium and large sized birds more frequently used mechanical cervical dislocation devices such as Livetec Nex®, which was not the case for individuals working with smaller birds (Table 2). We found greater variability in participant responses when working with medium and large sized birds compared to small birds, where a clear majority always use manual cervical dislocation. Instead, when working with medium and large birds respondents reported using various methods more infrequently (often, sometimes, and rarely) rather than reporting a designated method for continuous use.

**Table 2:** Number and percentage of participants in brackets of all those working with small (≤3 kg), medium (>3 to ≤5 kg) or large (>5 kg) birds and the frequency of application for each on-farm killing method.

Method	Application frequency	Small (≤3 kg) (n = 162)	Medium (>3 to ≤5 kg) (n = 25)	Large (>5 kg) (n = 28)
Manual cervical dislocation	Always	148 (91.4%)	7 (28.0%)	0 (0.0%)
	Often	9 (5.6%)	9 (36.0%)	20 (71.4%)
	Sometimes	2 (1.2%)	6 (24.0%)	6 (21.4%)
	Rarely	0 (0.0%)	3 (12.0%)	2 (7.1%)
	Never	3 (1.9%)	0 (0.0%)	0 (0.0%)
Mechanical cervical dislocation (broomstick)	Always	0 (0.0%)	0 (0.0%)	0 (0.0%)
	Often	0 (0.0%)	0 (0.0%)	0 (0.0%)
	Sometimes	2 (1.2%)	1 (4.0%)	0 (0.0%)
	Rarely	2 (1.2%)	4 (16.0%)	1 (3.6%)
	Never	158 (97.5%)	20 (80.0%)	27 (96.4%)
Mechanical cervical dislocation (pliers)	Always	0 (0.0%)	0 (0.0%)	0 (0.0%)
	Often	0 (0.0%)	0 (0.0%)	0 (0.0%)
	Sometimes	4 (2.5%)	2 (8.0%)	0 (0.0%)
	Rarely	1 (0.6%)	1 (4.0%)	0 (0.0%)
	Never	157 (96.9%)	22 (88.0%)	28 (100.0%)
Mechanical cervical dislocation (cone)	Always	0 (0.0%)	0 (0.0%)	0 (0.0%)
	Often	0 (0.0%)	0 (0.0%)	0 (0.0%)
	Sometimes	0 (0.0%)	0 (0.0%)	0 (0.0%)
	Rarely	0 (0.0%)	1 (4.0%)	0 (0.0%)
	Never	162 (100.0%)	24 (96.0%)	28 (100.0%)
Mechanical cervical dislocation (Livetec Nex®)	Always	0 (0.0%)	3 (12.0%)	3 (10.7%)
	Often	23 (14.2%)	8 (32.0%)	16 (57.1%)
	Sometimes	7 (4.3%)	2 (8.0%)	1 (3.6%)
	Rarely	16 (9.9%)	6 (24.0%)	0 (0.0%)
	Never	116 (71.6%)	6 (24.0%)	8 (28.6%)
Blunt force trauma	Always	0 (0.0%)	0 (0.0%)	0 (0.0%)
	Often	3 (1.9%)	0 (0.0%)	0 (0.0%)
	Sometimes	1 (0.6%)	0 (0.0%)	0 (0.0%)
	Rarely	0 (0.0%)	1 (4.0%)	0 (0.0%)
	Never	158 (97.5%)	24 (96.0%)	28 (100.0%)
Captive bolt (cartridge)	Always	1 (0.6%)	0 (0.0%)	0 (0.0%)
	Often	0 (0.0%)	1 (4.0%)	1 (3.6%)
	Sometimes	2 (1.2%)	12 (48.0%)	1 (3.6%)
	Rarely	6 (3.7%)	7 (28.0%)	26 (92.9%)
	Never	153 (94.4%)	5 (20.0%)	0 (0.0%)
Captive bolt (noncartridge)	Always	0 (0.0%)	0 (0.0%)	0 (0.0%)
	Often	1 (0.6%)	1 (4.0%)	0 (0.0%)
	Sometimes	1 (0.6%)	1 (4.0%)	0 (0.0%)
	Rarely	5 (3.1%)	0 (0.0%)	0 (0.0%)
	Never	155 (95.7%)	23 (92.0%)	28 (100.0%)
Decapitation	Always	2 (1.2%)	0 (0.0%)	0 (0.0%)
	Often	1 (0.6%)	0 (0.0%)	0 (0.0%)
	Sometimes	5 (3.1%)	0 (0.0%)	0 (0.0%)
	Rarely	2 (1.2%)	0 (0.0%)	0 (0.0%)
	Never	152 (93.8%)	25 (100.0%)	28 (100.0%)
Gas	Always	0 (0.0%)	0 (0.0%)	0 (0.0%)
	Often	0 (0.0%)	0 (0.0%)	0 (0.0%)
	Sometimes	4 (2.5%)	1 (4.0%)	0 (0.0%)
	Rarely	4 (2.5%)	0 (0.0%)	0 (0.0%)
	Never	154 (95.1%)	24 (96.0%)	28 (100.0%)
Electrical stun to kill	Always	0 (0.0%)	0 (0.0%)	0 (0.0%)
	Often	0 (0.0%)	0 (0.0%)	0 (0.0%)
	Sometimes	2 (1.2%)	1 (4.0%)	0 (0.0%)
	Rarely	2 (1.2%)	0 (0.0%)	0 (0.0%)
	Never	158 (97.5%)	24 (96.0%)	28 (100.0%)
Overdose of anesthetic	Always	0 (0.0%)	0 (0.0%)	0 (0.0%)
	Often	2 (1.2%)	1 (4.0%)	0 (0.0%)
	Sometimes	7 (4.3%)	2 (8.0%)	0 (0.0%)
	Rarely	10 (6.2%)	4 (16.0%)	0 (0.0%)
	Never	143 (88.3%)	18 (72.0%)	28 (100.0%)

### The Role of Confidence on the Use of on Farm Dispatching Methods

Participants were asked whether they were confident in applying/using each killing method. We found that 98.6% (n = 212) reported confidence in manual cervical dislocation, whereas less than half of participants reported confidence for all other methods (Table 3). We found that confidence depended upon availability for most methods, whereby participants were more likely to report confidence in a method if it were available at their current workplace (all *P*-values <0.05), except for mechanical cervical dislocation using a cone and captive bolt (noncartridge), whereby the likelihood of reporting confidence was unaffected by method availability (Z_ratio_ = 0.005, *P* = 0.996, Z_ratio_=0.009, *P* = 0.993 respectively).

**Table 3:** Percentage of participants in brackets reporting confidence, method availability and those who stated “always used” for each killing method out of a total 215 responses.

Method	Confidence (%)	Availability (%)	“Always” use (%)
Manual cervical dislocation	98.6	100.0	72.1
Mechanical cervical dislocation (broomstick)	4.2	4.2	0.0
Mechanical cervical dislocation (pliers)	7.0	2.8	0.0
Mechanical cervical dislocation (cone)	0.9	0.5	0.0
Mechanical cervical dislocation (Livetec Nex®)	32.6	42.8	2.8
Blunt force trauma	2.8	26.0	0.0
Overdose of anesthetic	10.7	8.4	0.0
Captive bolt (cartridge)	24.7	24.7	0.5
Captive bolt (noncartridge)	4.2	1.4	0.0
Decapitation	4.2	3.3	0.9
Electrical stun to kill	2.8	1.9	0.0
Gas	2.8	2.3	0.0

We investigated whether bird size (small [<3 kg], medium [3–5 kg], or large [>5 kg]) had any influence on individuals’ likelihood of reporting confidence in a method. We found no influence of bird size on the likelihood of reporting confidence in manual cervical dislocation, decapitation, gas, blunt force trauma or mechanical cervical dislocation using a broomstick, pliers, or cone. However we did find an effect of bird size on the likelihood of reporting confidence when considering Livetec Nex® (X_2(2)_ = 31.1, *P* < 0.001). Participants were more likely to be confident using Nex when working with medium or large birds compared to small birds (Z_ratio_ = 2.57, *P* = 0.027 and Z_ratio_ = 4.88, *P* < 0.001 respectively). Participants were more likely to be confident with overdose of anesthetic when working with medium sized birds compared to small birds (Z_ratio_ = 2.57, *P* = 0.027). This was also the case when considering confidence in utilizing electrical stun to kill (Z_ratio_ = 3.04, *P* = 0.0067) and cartridge captive bolt guns (Z_ratio_ =6.61, *P* < 0.001). Finally, we found greater confidence in noncartridge captive bolt guns in participants primarily working with larger birds compared to individuals working with small birds (Z_ratio_ = 3.30, *P* = 0.0028).

### Method Preference and Properties Behind Killing Method Selection

Participants were asked to indicate their personal preferred method to kill an individual bird. The most widely selected method was manual cervical dislocation (81.9%, n = 176), followed by mechanical cervical dislocation using Livetec Nex® (10.2%, n = 22), then captive bolt gun (cartridge) (6.5%, n = 14) with only a few respondents selecting decapitation (0.5%, n = 1) and overdose of anesthetic (0.5%, n = 1) or other (0.5%, n = 1).

**Figure 2:**
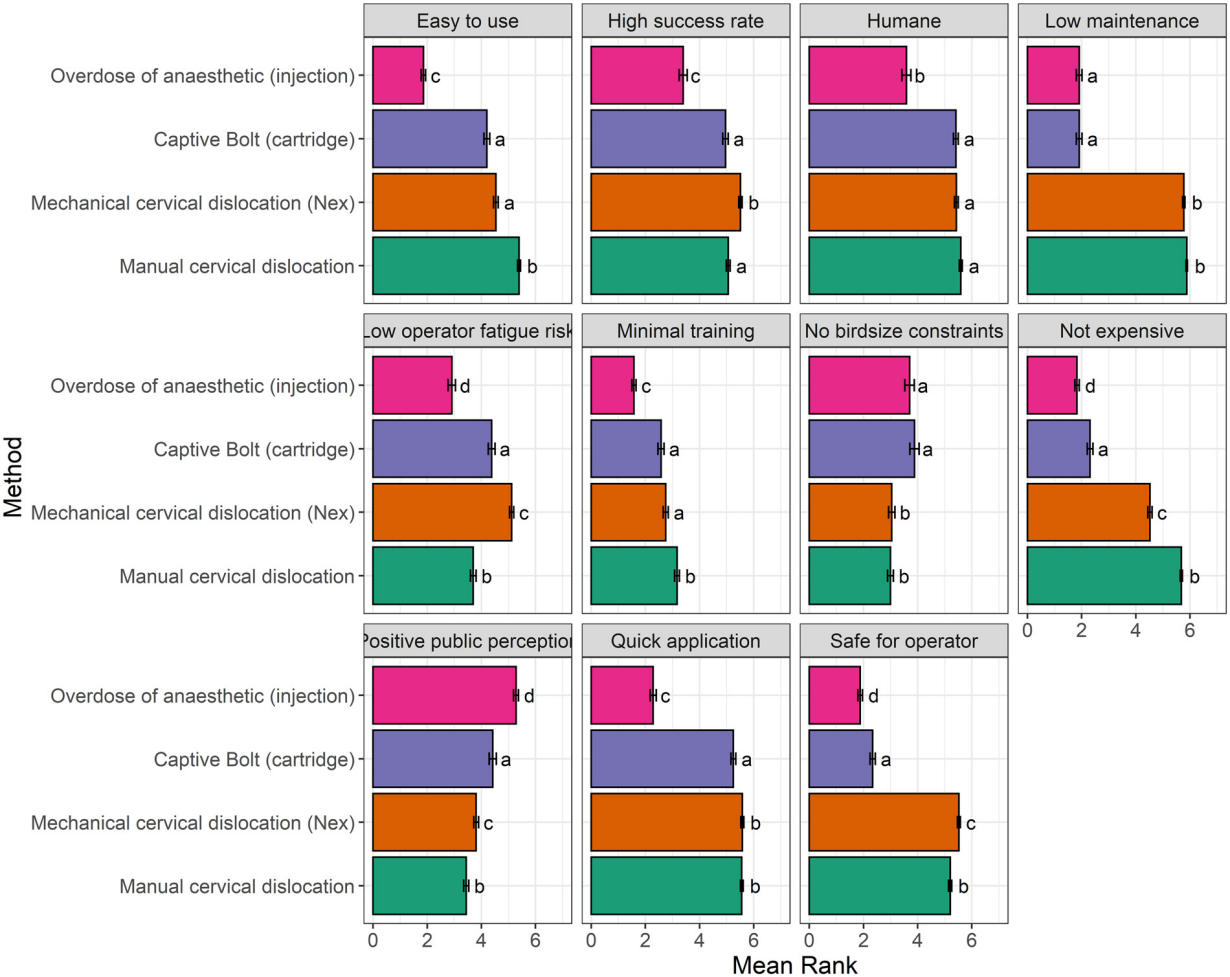
Mean ± SE scores from Likert scale for a range of killing method properties across each killing method. Participants were asked to rate each property according to the following scale: (1 = strongly disagree; 2 = moderately disagree; 3 = slightly disagree; 4 = slightly agree; 5 = moderately agree; 6 = strongly agree). Difference in letters denote statistical significance (*P* < 0.05) between groups.

All respondents (n = 215) were then asked if they agreed or disagreed with each statement when considering 4 selected killing methods (overdose of anesthetic, decapitation, manual cervical dislocation and Nex) in turn (Figure 2). Overall agreement as to whether a method was humane, showed manual cervical dislocation (91.6%, n = 197), mechanical cervical dislocation using Nex (79.0%, n = 170) and captive bolt (46.9%, n = 101) with the highest agreement scores (respondents agreeing or strongly agreeing). In contrast overdose of anesthetic was shown to be lowest when compared to all other methods (captive bolt: Z_ratio_ = 9.57, *P* < 0.0001; manual cervical dislocation: Z_ratio_ = 11.32, *P* < 0.0001; Nex: Z_ratio_ = 10.11, *P* < 0.0001) with (22.8%, n = 49) disagreeing or strongly disagreeing. We also found a higher degree of disagreement with overdose of anesthetic being easy to use with 40.5% (n = 87) disagreeing or strongly disagreeing compared to 32.6% (n = 70) for captive bolt (Z_ratio_ = 16.95, *P* < 0.0001), 0.5% (n = 1) for manual cervical dislocation (Z_ratio_=34.92, *P* < 0.0001) and 0% for Nex (Z_ratio_ = 22.23, *P* < 0.0001). However, both captive bolt and Nex were considered less easy to use compared to manual cervical dislocation (Z_ratio_ = 9.79, *P* < 0.0001, Z_ratio_ = 8.46, *P* < 0.0001 respectively). In terms of a method's ability to be successful, Nex was considered to have the greatest success with 74.9% (n = 161) individuals agreeing, closely followed by manual cervical dislocation with 74% (n = 159) agreeing, followed by captive bolt with 40.5% (n = 87) agreeing and lastly overdose of anesthetic which had the lowest agreement of all methods for its ability to provide a successful method (captive bolt: Z_ratio_ = 8.75, *P* < 0.0001, manual cervical dislocation: Z_ratio_ = 10.09, *P* < 0.0001, Nex: Z_ratio_ = 13.20, *P* < 0.0001). A method's ability to provide a quick application was also ranked, overall agreement showed that all methods were ranked higher than overdose of anesthetic (captive bolt: Z_ratio_ = 20.06, *P* < 0.0001; manual cervical dislocation: Z_ratio_ = 26.11, *P* < 0.0001; Nex: Z_ratio_ = 25.63, *P* < 0.0001). Manual cervical dislocation yielded the highest agreement score with 99.5% (n = 214) of respondents agreeing or strongly agreeing that it was quick to apply. There was also high agreement for Nex (72.5%, n = 156) and captive bolt with 40% (n = 86) agreeing or strongly agreeing but this was lower than agreement for both manual cervical dislocation (Z_ratio_ = 3.05, *P* = 0.012) and Nex (Z_ratio_= 3.06, *P* = 0.012). However, the lowest agreement was obtained for overdose of anesthetic where more individuals disagreed with its ability to be quick with 33% (n = 71) disagreeing or strongly disagreeing. Expense was another important consideration, where overdose of anesthetic was ranked lowest in terms of its ability to be low cost compared to all other methods (captive bolt: Z_ratio_ = 3.58, *P* = 0.0019; manual cervical dislocation: Z_ratio_ = 41.22, *P* < 0.0001; Nex: Z_ratio_=23.45, *P* < 0.0001), with 42.8% (n = 92) of individuals disagreeing or strongly disagreeing with this statement. This contrasts to manual cervical dislocation (98.6%, n = 212), mechanical cervical dislocation using Nex (44.2%, n = 95) and captive bolt (2.8%, n = 6) where individuals agreed that they were lower cost than overdose of anesthetic. There was lower agreement when considering captive bolt compared to Nex (Z_ratio_ = 16.95, *P* < 0.0001) and manual cervical dislocation (Z_ratio_ = 29.99, *P* < 0.0001) and more individuals disagreed with the capacity of mechanical cervical dislocation as being low cost compared to manual cervical dislocation (Z_ratio_ = 13.27, *P* < 0.0001).

With regards to operator-based factors, we found that 40.5% (n = 87) of individuals disagreed that overdose of anesthetic was safe for the operator compared to all other methods (captive bolt: Z_ratio_ = 3.63, *P* = 0.0016; manual cervical dislocation: Z_ratio_ = 31.76, *P* < 0.0001; Nex: Zratio = 35.87, *P* < 0.0001). In terms of safety, 32.6% (n = 70) of respondents disagreed that captive bolt was safe compared to manual cervical dislocation (3.3%, n = 7 disagreed; Z_ratio_ = 25.33, *P* < 0.0001) and Nex (0% disagreed; Z_ratio_ = 28.88, *P* < 0.0001). Furthermore, more individuals agreed that Nex was safe compared to manual cervical dislocation (Z_ratio_ = 4.00, *P* < 0.0001). Therefore, Nex was associated with the greatest agreement and considered the safest compared to all other methods (Figure 2). Equipment maintenance is also a consideration when utilizing on-farm killing methods and therefore personnel were asked to rate each method according to whether it is low maintenance to use/operate. We found high agreement scores for both manual and mechanical (Nex) cervical dislocation with 99.5% (n = 214) and 78.6% (n = 169) agreeing with these methods being low maintenance. This contrasts to overdose of anesthetic (manual cervical dislocation: Z_ratio_ = 35.79, *P* < 0.0001; Nex: Z_ratio_ = 33.14, *P* < 0.0001) and captive bolt (manual cervical dislocation: Z_ratio_= 35.93, *P* < 0.0001; Nex: Z_ratio_ = 33.26, *P* < 0.0001), where 41.4% (n = 89) and 37.7% (n = 81) disagreed that these methods were low maintenance. Operator fatigue has been a significant concern over recent years and therefore individuals were asked to rate their agreement for each method being associated with a low risk of operator fatigue. We found that mechanical cervical dislocation using Nex had the greatest agreement with 63.3% (n = 136) of individuals agreeing with it being associated with a low risk of operator fatigue. This contrasted with manual cervical dislocation (Z_ratio_=10.88, *P* < 0.0001) and captive bolt (Z_ratio_=4.98, *P* < 0.0001) where only 34% (n = 73) and 27.9% (n = 60) of individuals agreed respectively. However, overdose of anesthetic was ranked the lowest compared to all other methods (captive bolt: Z_ratio_ = 7.95, *P* < 0.0001; manual cervical dislocation: Z_ratio_= 4.61, *P* < 0.0001; Nex: Z_ratio_ = 13.94, *P* < 0.0001), whereby only 13.1% (n = 28) of respondents agreed that this method was associated with a low risk of operator fatigue. The requirement for minimal training was also assessed, the method with highest disagreement was overdose of anesthetic where 46% (n = 99) of respondents disagreed or strongly disagreed that this method required minimal training. This contrasted with all other methods, including captive bolt (Z_ratio_= 7.31, *P* < 0.0001) where 26.5% (n = 57) disagreed, manual cervical dislocation (Z_ratio_ = 13.48, *P* < 0.0001) where 36.2% (n = 78) of individuals disagreed, and Nex (Z_ratio_=9.97, *P* < 0.0001) where 40.9% (n = 88) of individuals disagreed. More individuals disagreed with Nex and captive bolt requiring minimal training compared to manual cervical dislocation (Z_ratio_ = 3.06, *P* = 0.012 and Z_ratio_ = 4.10, *P* = 0.0002 respectively).

When considering recent legislative reform, we also asked individuals about bird size/weight constraints on their method choice. We found more individuals agreed with lack of bird size constraints when considering overdose of anesthetic, where 27.5% (n = 59) agreed or strongly agreed with this statement. This contrasted with mechanical cervical dislocation via Nex (13.0%, n = 28 agreed, Z_ratio_ = 3.12, *P* < 0.001) and manual cervical dislocation (14.0%, n = 30 agreed, Z_ratio_ = 3.40, *P* = 0.0038) but was similar to captive bolt (22%, n = 48 agreed, Z_ratio_ = 0.75, *P* = 0.877). Similarly, captive bolt had higher agreement than both mechanical cervical dislocation (Nex) (Z_ratio_ = 4.06, *P* = 0.0003) and manual cervical dislocation (Z_ratio_ = 4.36, *P* = 0.0001) which had the lowest mean agreement score (Figure 2). Overdose of anesthetic was considered to have a more positive public perception than all other methods (captive bolt: Z_ratio_ = -5.39, *P* < 0.0001; manual cervical dislocation: Z_ratio_ = -14.33, *P* < 0.0001; Nex: Z_ratio_ = -11.53, *P* < 0.0001). With regards to overdose of anesthetic, 44.2% (n = 95) of individuals agreed with this statement compared to only 23.3% (n = 50) for captive bolt, 22.8% (n = 49) for manual cervical dislocation and 12.1% (n = 26) for Nex. However, when looking at the mean scores (Figure 2) individuals showed greater agreement with this statement when considering Nex compared to manual cervical dislocation (Z_ratio_ = 2.79, *P* = 0.027) and when considering captive bolt compared to both manual (Z_ratio_=6.03, P < 0.001) and mechanical cervical dislocation (Z_ratio_=3.79, *P* = 0.0009).

We were interested in determining the importance of various properties more generally when selecting a killing method of choice. Therefore, participants were asked to rank a range of killing method properties, irrespective of method, based on their level of importance with 1 representing ‘no importance’ and 10 being the ‘most important’. Overall, a methods ability to be effective was ranked as the most important, closely followed by a methods ability to be humane (Table 4).

**Table 4:** Mean rank and 95% confidence intervals in ascending order for killing method properties irrespective of method and demographic factors, based on their level of importance (1 = “no importance” to 10 = “most important”).

Property	Mean rank	95% CIs
Positive public perception	5.82	5.44–6.20
Not expensive	6.58	6.23–6.93
Low operator fatigue	6.71	6.39–7.04
Minimal training^1^	6.78	6.40–6.96
No equipment	6.89	6.56–7.22
Low maintenance	7.03	6.73–7.33
Safe for the operator^1^	8.25	8.01–8.48
Easy to use	8.44	8.25–8.63
Quick application	9.12	9.01–9.24
Humane^1^	9.30	9.18–9.43
High success rate^1^	9.79	9.73–7.85

1 denotes properties where statistical modeling was unfeasible due to lack of diversity in the data.

**Figure 3:**
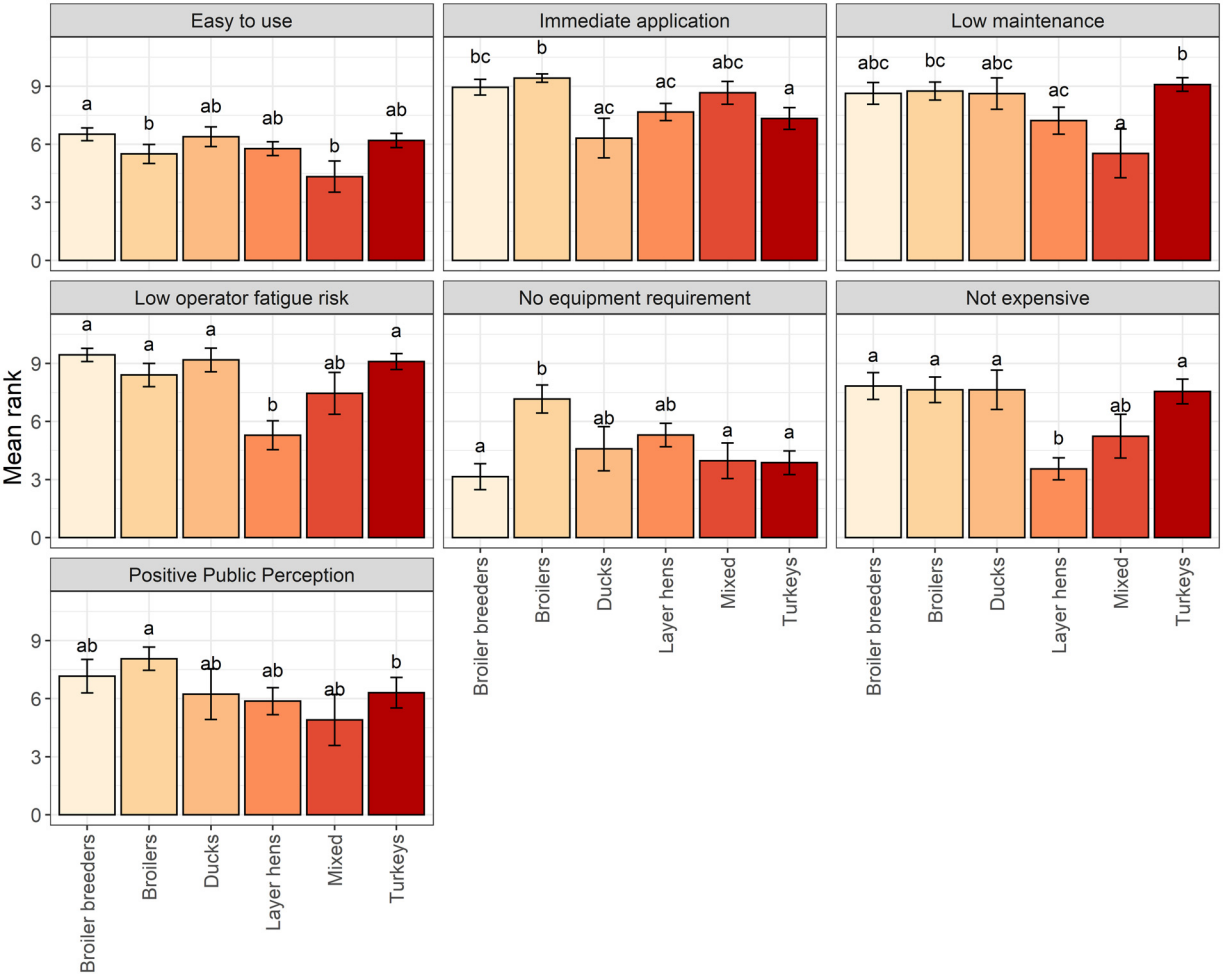
Mean ± SE scores for a range of killing method properties according to primary species. Participants were asked to rate each property according to the level of importance with rank 1 being of no importance and rank 10 being the most important. Difference in letters denote statistical significance (*P* < 0.05) between groups.

However, the ranking of these properties was influenced by the species respondents primarily worked with (Figure 3). Participants were more likely to rank cost as less important when working with layer hens than all other species (broiler breeders: Z_ratio_ = 5.64, *P* < 0.0001; broilers: Z_ratio_ = 5.91, *P* < 0.0001; ducks: Z_ratio_ = 3.96, *P* = 0.001; turkeys: Z_ratio_ = 6.31, *P* < 0.0001). Ease of application was more likely to be ranked as less important for participants primarily working with mixed species compared to broiler breeders (Z_ratio_ = 3.10, *P* = 0.02), and for broilers compared to broiler breeders (Z_ratio_ = 3.32, *P* = 0.012). The importance of immediate application was ranked highly regardless of demographic factors; however, it was ranked higher when working with broilers compared to turkeys (Z_ratio_ = 4.91, *P* < 0.0001), ducks (Z_ratio_ = 3.36, *P* = 0.01) or layer hens (Z_ratio_ = 3.44, *P* = 0.0078) and when working with broiler breeders compared to turkeys (Z_ratio_ = 3.51, *P* = 0.006). A methods ability to be low maintenance and association with low operator fatigue were also considered highly important overall. However, maintenance of equipment was deemed more important when working with turkeys compared to mixed species (Z_ratio_ = 3.05, *P* = 0.028) and laying hens (Z_ratio_ = 3.16, *P* = 0.02), and when working with broilers compared to mixed species (Z_ratio_ = 2.92, *P* = 0.04). Low risk of operator fatigue was considered less important for method selection when working with layer hens compared to broiler breeders (Z_ratio_ = 5.98, *P* < 0.0001), broilers (Z_ratio_ = 4.36, *P* = 0.0002), turkeys (Z_ratio_ = 6.12, *P* < 0.0001), and ducks (Z_ratio_ = 4.90, *P* < 0.0001). The requirements for no equipment was deemed more important when working with broilers compared to broiler breeders (Z_ratio_ = 7.38 *P* < 0.0001), mixed species (Z_ratio_ = 3.75, *P* = 0.0024), and turkeys (Z_ratio_ = 6.80, *P* < 0.0001). Finally positive public perception was found to have a role in method selection, whereby participants working with broilers were more likely to rank positive public perception higher than those working with turkeys (Z_ratio_ =3 .86, *P* = 0.0016).

Other factors such as sector and farming system were also found to influence the importance of numerous killing method properties. Cost was more important for participants working in indoor noncaged farming systems compared to those working in mixed farming systems (mean: 5.21 ± 0.382 vs. 8.45 ± 0.832, Z_ratio_ = 3.58, *P* = 0.0032). Public perception was more important in the breeding sector compared to ‘other’ (mean: 8.37 ± 1.26 vs. 4.36 ± 0.944, Z_ratio_ = 2.77, *P* = 0.0287) and was more important for those working in veterinary services (mean: 7.41 ± 0.655) compared to other (4.36 ± 0.944, Z_ratio_ = 3.54, *P* = 0.0022) and producers (mean: 5.54 ± 0.638, Z_ratio_ = 3.92, *P* = 0.0005).

### Reasons for Killing an Individual Bird on Farm

**Figure 4:**
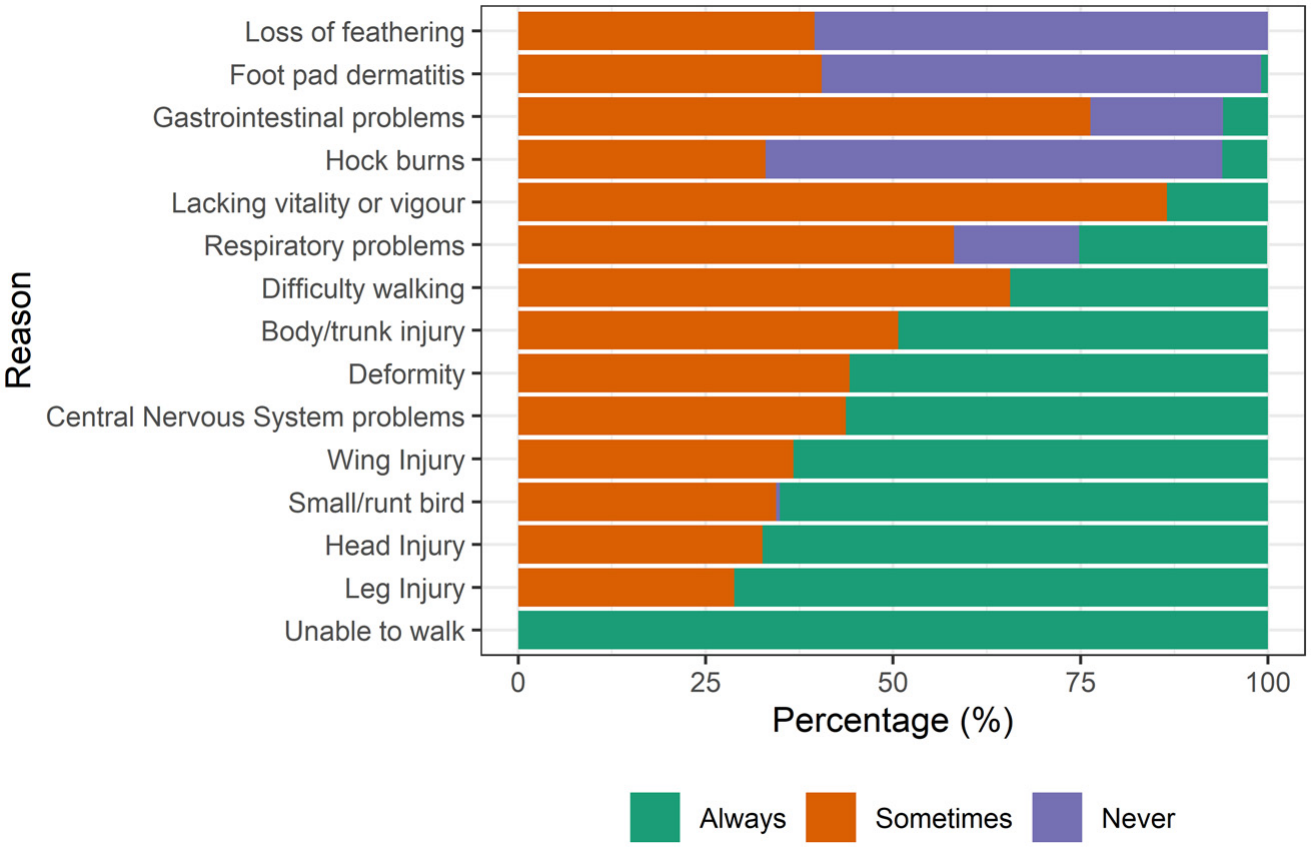
Mean percentage of participants stating requirement for on farm kill according to a range of health/ clinical conditions, options always, sometimes, or never were allowed.

Participants were asked which reasons would require an individual bird to be killed on farm. The only factor with 100% agreement if the bird was unable to walk, where all participants stated that they would “always” kill the bird (Figure 4). In comparison, other factors such as respiratory problems, loss of feathering, foot pad dermatitis, hock burns and gastrointestinal problems resulted in inconsistent responses ranging from “never” to “always” (Figure 4). All other health conditions were reported to be either “sometimes” or “always” employed for killing an individual bird. Demographic factors such as primary species, sector or farming system had no influence on the health conditions/reasons that would require an individual bird to be dispatched on farm.

### Attitudes Towards Current Legislation in the UK

Participants were provided with a range of statements in relation to the legislative changes associated with the enforcement of Council Regulation (EC) No. 1099/2009 in 2013 across the EU and UK surrounding the use of manual and mechanical cervical dislocation. Participants were asked to indicate their extent of agreement or disagreement with each statement (Table 5). In relation to bird welfare, most participants agreed to some extent (75.3%, n = 162) that limiting manual cervical dislocation according to bird weight protects bird welfare. However, this was not the case when considering restricting the number of birds that manual cervical dislocation could be applied to by a single operator daily, where just over half of all participants (53.6%, n = 115) disagreed that this would protect welfare. In terms of legislation aimed at limiting operator fatigue, most individuals agreed that limiting the number of birds that manual cervical dislocation can be performed on (74%, n = 159) and that using bird weight (75.3%, n = 162) helps protect against operator fatigue. Despite this, most participants disagreed with the inclusion of weight limits in legislation, with 60.5% (n = 130) disagreeing that a weight limit of 3 kg for manual cervical dislocation is acceptable and 60.5% (n = 130) disagreeing with a 5 kg weight limit for mechanical cervical dislocation. Similarly, participants generally disagreed with the restriction of applying manual cervical dislocation to 70 birds per person per day (56.3%, n = 121). Finally, participants were asked about legislative weight limits and their impact on kill method choice. When considering a weight limit of 3 kg for manual cervical dislocation, just over half of all participants (56.8%, n = 122) did not consider this to influence method choice. In contrast, 65.7% (n = 141) of participants agreed that a 5 kg weight limit for mechanical cervical dislocation influenced their dispatching method choice.

**Table 5:** Breakdown of the number and percentage (%) in brackets of 215 participants according to agreement with various statements.

Statement	Strongly disagree	Moderately disagree	Slightly disagree	Slightly agree	Moderately agree	Strongly agree
Limiting manual cervical dislocation by bird weight protects bird welfare.	7 (3.3%)	16 (7.4%)	30 (14.0%)	40 (18.6%)	85 (39.5%)	37 (17.2%)
Restricting the number of birds manual cervical dislocation can be performed on daily protects bird welfare.	30 (14.0%)	38 (17.7%)	47 (21.9%)	57 (26.5%)	32 (14.9%)	11 (5.1%)
Limiting manual cervical dislocation by bird weight protects against operator fatigue.	4 (1.9%)	18 (8.4%)	31 (14.4%)	63 (29.3%)	80 (37.2%)	19 (8.8%)
Restricting the number of birds manual cervical dislocation can be performed on daily protects against operator fatigue.	6 (2.8%)	16 (7.4%)	34 (15.8%)	46 (21.4%)	75 (34.9%)	38 (17.7%)
A weight limit of 3 kg for manual cervical dislocation is acceptable.	47 (21.9%)	17 (7.9%)	66 (30.7%)	41 (19.1%)	18 (8.4%)	26 (12.1%)
The restriction of applying manual cervical dislocation to 70 birds per person per day is acceptable.	59 (27.4%)	30 (14.0%)	32 (14.9%)	67 (31.2%)	22 (10.2%)	5 (2.3%)
The weight limit of 3 kg for manual cervical dislocation impacts dispatching method choice.	52 (24.2%)	47 (21.9%)	23 (10.7%)	33 (15.3%)	33 (15.3%)	27 (12.6%)
The weight limit of 5 kg for mechanical cervical dislocation impacts dispatching method choice.	4 (1.9%)	27 (12.6%)	43 (20.0%)	50 (23.3%)	38 (17.7%)	53 (24.7%)
A weight limit of 5 kg for mechanical cervical dislocation is acceptable.	46 (21.4%)	37 (17.2%)	47 (21.9%)	46 (21.4%)	20 (9.3%)	19 (8.8%)

**Figure 5:**
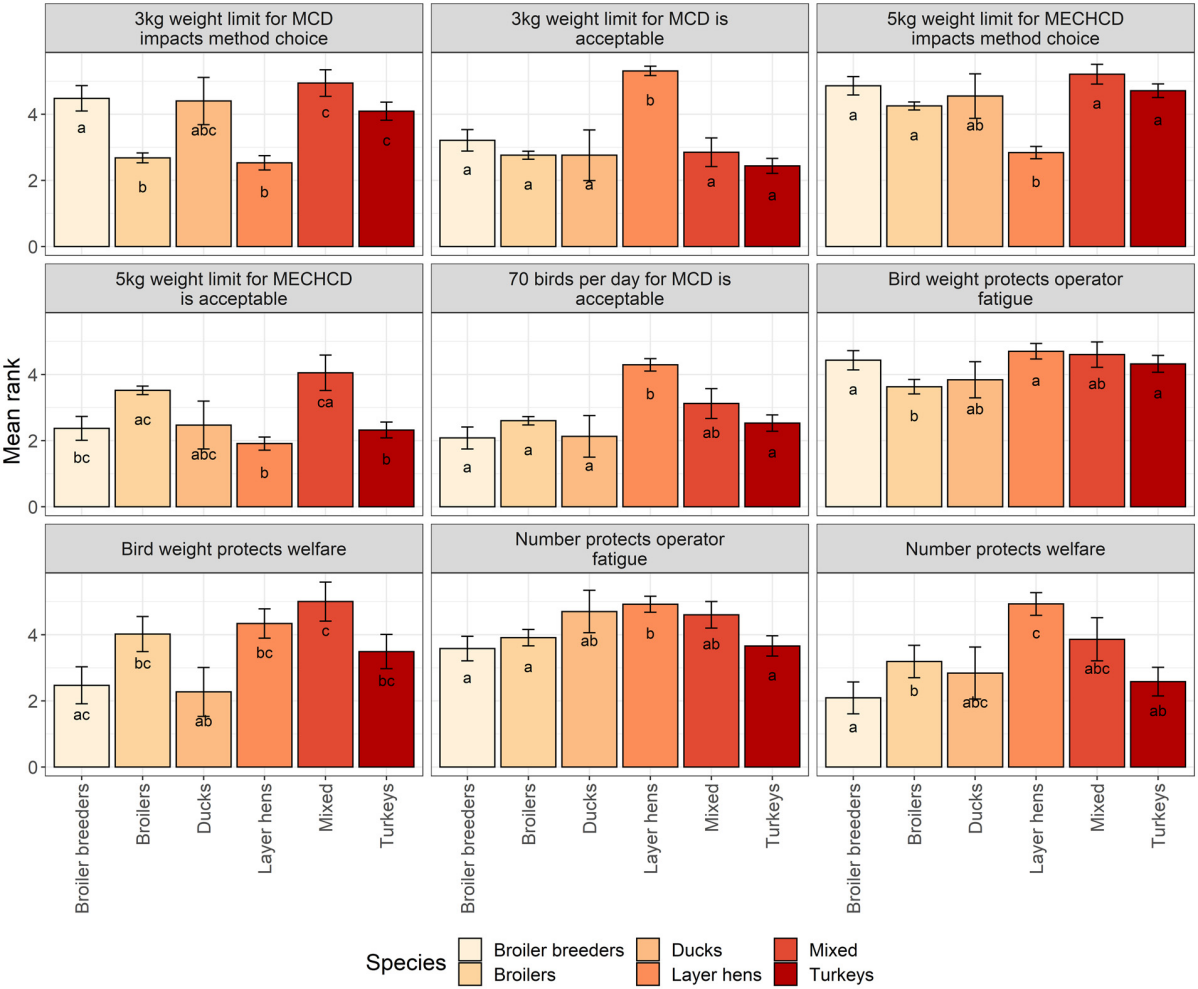
Mean ± SE scores for statements according to participants’ primary species. Participants were asked to rate each statement according to the following scale: (1 = strongly disagree; 2 = moderately disagree; 3 = slightly disagree; 4 = slightly agree; 5 = moderately agree; 6 = strongly agree). Difference in letters denote statistical significance (*P* < 0.05) between groups.

Primary species influenced attitudes towards existing legislation (Figure 5). Generally, those working primarily with broiler breeders were more likely to disagree with the statements provided compared to those working with other species and agreed that weight limits impacted on method choice. Individuals working with broiler breeders were more likely to disagree with the following statements: limiting manual cervical dislocation by bird weight protects bird welfare, restricting the number of birds manual cervical dislocation can be performed on daily protects bird welfare and restricting the number of birds manual cervical dislocation can be performed on daily protects against operator fatigue compared to those working with other species (Figure 5). However, this was not the case when considering bird weight limits protecting operator fatigue, where instead those working with broiler breeders were more likely to agree compared to those working with broilers. In general, personnel working primarily with laying hens were more likely to agree with most statements provided reflecting legislative changes especially when considering the 3 kg weight limit being acceptable and the restriction of applying manual cervical dislocation to 70 birds per person per day (Figure 5).

## DISCUSSION

Our findings provide essential information regarding the availability and use of on-farm killing methods for killing commercial poultry across the UK for the first time since legal reform in 2013 to Council Regulation (EC) No. 1099/2009 (EC, 2009) and the UK national regulations that enforce PATOK, The Welfare of Animals at the Time of Killing Regulations (England 2015; Scotland 2012; Wales and Northern Ireland 2014 (WATOK) (UK Government, 2015)). In addition, these findings provide valuable insight into the perspectives of stock-workers utilizing them, and their attitudes towards the addition of restrictions on the number of birds and bird weight limits in legislation. Furthermore, our results demonstrate the priorities of stock-workers in killing method selection, which could provide critical insights for the future development or refinement of on-farm killing methods and highlight the criticisms for alternative methods developed to replace manual cervical dislocation (e.g., Livetec Nex® and captive bolt). In line with previous findings (Martin, 2015; Watteyn et al., 2020), we demonstrate that despite legal reform, manual cervical dislocation remains the most prevalent method used across the UK for killing poultry on farm (with 100% of participants stating it was available to them) and remains the preferred method amongst respondents (81.9%). Our work was not limited by a small sample size and builds on those previously reported (Watteyn et al., 2020), however like any anonymous survey dealing with sensitive data we cannot fully rule out nonindependence of participant responses. We demonstrate that the primary species personnel work with, and thus bird weight, impacted method availability, application frequency, reported confidence and individual attitudes towards killing and legislative reform.

Manual cervical dislocation was available to all participants regardless of the species they primarily work with, and bird size did not affect individuals’ confidence in performing manual cervical dislocation. In contrast, mechanical cervical dislocation was only available to approximately half of participants but was more widely available to individuals working with birds >3 kg, perhaps as a consequence of legislative reform and no legal requirement to utilize these methods. However, our findings suggest that although individuals working with medium (>3 to ≤5 kg) or large birds (>5 kg) have greater access to mechanical cervical dislocation methods, they may not consistently utilize them when killing an individual bird on farm. Some individuals continue to employ manual cervical dislocation to birds classified in the >3 kg group despite the weight restrictions added to legislation, and therefore our findings may suggest that reform has not prevented cervical dislocation being used as a routine method for killing poultry on farm for birds over 3 kg. However, it is important to note that the bird size classifications set in this study, grouped stock-workers based on the type of poultry they work with and the average final live weight of the birds at slaughter/end of production. As a result, turkeys were classified as large (>5 kg), however in general turkeys do not reach >5 kg body weight until after 12 wk of age (with variation based on sex (e.g., Aviagen Turkeys Limited, 2015)). Therefore, for the first ∼12 wk cervical dislocation could be legally used and may explain why stock-workers working with medium or large birds reported having access and utilizing both manual and mechanical cervical dislocation. We found no clear mechanical method that individuals stated they “always” employ as an alternative when killing a bird >3 kg. Instead, we found a diverse range of responses reflecting less frequent use (i.e., often, sometimes, rarely) of mechanical methods, suggesting much more ambiguity in killing method selection and application when working with larger species. Perhaps the greater range of killing methods available, and being utilized by stock-workers, highlights consideration of altering killing method selection as the birds’ grow. However, there does not appear to be a standard repertoire of killing methods dependent on bird type, suggesting a lack of a gold standard methodology, which could risk bird welfare at killing as well as contributing to inconsistency and/or agreement within and across the industry. Given the high agreement in ranking killing method properties with the 2 top properties being “humane” and “high success rate,” the lack of consistency in methods used is concerning and suggests a lack of consensus or knowledge in the efficacy and the humaneness of the killing methods available. Scientific studies have suggested that application of the original Livetec Nex® prototype resulted in greater suppression of electrical brain activity immediately post application and resulted in a faster onset to isoelectric electroencephalogram activity than manual cervical dislocation (Martin et al., 2019). In addition, other studies have highlighted captive bolt resulted in quicker cessation of reflexes than manual cervical dislocation (Martin et al., 2018b; Baker-Cook et al., 2021b). However, other studies have shown that other forms of mechanical cervical dislocation (e.g., Koechner Euthanasia Device) and other types of captive bolt are less humane than manual cervical dislocation (Bader et al., 2014; Martin et al., 2016; Bandara et al., 2019b; Baker-Cook et al., 2021b). The diversity in method performance highlights the issues with solely grouping killing devices based on their intended purpose. Captive bolt devices can vary greatly dependent upon factors such as bolt size and shape, power, bolt velocity etc., as well as their potential for mechanical fault such as misfiring or jamming (Martin et al., 2018b; Baker-Cook et al., 2021b). Mechanical cervical dislocation methods also vary based on their intended dislocation target location, stretch and/or twist operation, and crushing risk etc. (Sparrey et al., 2014; Martin et al., 2018a). Therefore, we intentionally separated mechanical cervical dislocation according to different techniques in our survey given the variation in application and outcome (e.g., increased risk of crushing and death via asphyxiation when utilizing broomsticks and pliers (Sparrey et al., 2014; Martin et al., 2017)), as this may influence bird care staff method/technique selection. This highlights that generalization is dangerous and poses a serious risk to bird welfare. Additionally, whilst there is scientific evidence relating to many of the killing methods available on farm for poultry, this knowledge may not be translating into practice and may not be easily accessible to the industry and specifically to those individuals utilizing these methods. For example, the stock-workers surveyed in this study reported that they believed manual cervical dislocation, captive bolt and the Livetec Nex® were equally humane. This highlights the importance of applied research being made accessible to relevant stakeholders (Graham et al., 2006), especially given the outcomes of killing method selection play a major part in protecting poultry welfare on-farm. Additionally, it further highlights that methods such as the CASH Small Animal Tool (Frontmatec Accles & Shelvoke, 2022), which have been evidenced as high welfare (Martin et al., 2018b; Gibson et al., 2019) and available since the legislation reform have not been taken up by the industry. This could be partly due to operator perceptions of challenges around operation, maintenance and technical issues raised following repeated use (Martin et al., 2018b). This raises the importance of engaging relevant stakeholders early in novel method development to inform design and operational needs, as well as to facilitate human behavior change leading to successful implementation of new products/methods which could enhance animal welfare (Purwins and Schulze-Ehlers, 2018; Balzani and Hanlon, 2020).

Understanding reasons behind method selection is important when bringing about changes to best practice and policy. Therefore, we asked participants to rate a range of properties when considering each method. A methods ability to provide an effective death was regarded the most important property amongst respondents. However, the way that properties were ranked was highly dependent upon the species that individuals predominantly worked with. This is likely reflective of the different housing systems and prevalence of health conditions amongst different species requiring on-farm killing. Broilers are by far the most widely farmed terrestrial species for meat production worldwide, with an estimated 72 billion broilers farmed globally each year (Food and Agriculture Organization of the United Nations (FAO), 2020), and as such are subject to great scrutiny and negative public attention. However, given that the slaughter weight of broilers is typically less than 3 kg (Department for Environment Food & Rural Affairs Defra, 2021), most birds that may require being killed on farm prior to slaughter is unaffected by the legislative weight limits for manual cervical dislocation. Therefore, our findings that stock-people working with broilers consider public perception, a lack of dedicated equipment and a methods ability to be low maintenance as more important, compared to those working with other species likely reflects their current experiences and practices. Anything other than manual cervical dislocation requires specialist equipment and requires more maintenance which is not currently part of their daily considerations. This contrasts with individuals working with larger birds (>3 kg), who are generally used to employing mechanical cervical dislocation and therefore consider these factors as less important or less of a barrier. Generally, we found that overdose of anesthetic was ranked poorly in terms of its ability to provide a humane, fast, and cost-effective death. However, it was ranked the highest out of the methods in terms of having a positive public perception. This is likely explained due to existing euthanasia practices for domestic and companion animals and public attitudes towards veterinarians as trusted and well-respected individuals (Mills, 2018; Alonso et al., 2020). However, in practice on a poultry farm this method often requires additional manual restraint, preparing equipment and pharmacologic agents and the delay results in longer times to loss of consciousness, which explains the disparity between public perception and responders’ negative scores for many of the practical properties for this method compared to manual cervical dislocation. The second most used and available method (manual cervical dislocation being the most used) was mechanical cervical dislocation using Livetec Nex® with 42.8% of respondents having access to it. Livetec Nex® was made available in 2018 to provide a way of mechanically dislocating birds weighing ≤5 kg in response to the weight restrictions added to legislation (Martin et al., 2016,^2019^; Livetec Systems Ltd., 2018). Certain properties were scored similar to manual cervical dislocation such as its ability to provide a humane and fast death. However, participants recognized it as being safer and easier, as well as its association with lower risk of operator fatigue and more positive public perception compared to manual cervical dislocation. While Livetec Nex® is a good alternative to manual cervical dislocation according to the property rankings, it is preferred by and associated with greater confidence in responders working with larger bird species (turkeys, broiler breeders, ducks, and mixed) likely due to: greater availability, weight limits placed on manual cervical dislocation, and potentially due to a preference by stock-workers to use this method on birds which (irrespective of the legal framework) are more challenging to cervically dislocate by hand. If, however Livetec Nex® was purchased by more farms and thus more widely available, responders working with smaller birds may choose this method over manual cervical dislocation, which would build confidence, could help protect against operator fatigue (Martin et al., 2018a) and improve bird welfare (Martin et al., 2016,^2019^). Like manual cervical dislocation though, mechanical cervical dislocation (Livetec Nex®) is perceived to have a negative public perception by poultry stock-workers, requires training, and has bird size constraints (restricted to birds weighing a maximum of 5 kg), which could act as barriers to its uptake.

In line with the Flemish study (Watteyn et al., 2020), we asked participants to state reasons that would require an individual bird to be killed on farm. It is well documented that broilers suffer from a range of health conditions as a consequence of intensive farming practices (Julian, 1998; Bessei, 2006; Dinev, 2012; OIE (World Animal Health Organization), 2019; Abeyesinghe et al., 2021), and as such, we were interested in determining whether there were differences in the reasons behind killing according to the species individuals predominantly worked with. The only reason with unanimous agreement was if a bird was unable to walk where all participants stated that they would “always” kill the bird. In comparison, other factors such as respiratory problems, loss of feathering, foot pad dermatitis, hock burns and gastrointestinal problems resulted in inconsistent responses ranging from “never” to “always” but were all unaffected by species. This is likely due to limitations in our survey design; although we expanded the list of reasons provided to participants compared to previous work (Watteyn et al., 2020), a finite list of common reasons were provided but with no reference to severity classification. Therefore, it is possible that this type of question created ambiguity and relied on the knowledge and experience of the operator to understand bird health and welfare to make informed decisions about whether killing was a necessary requirement.

Since the EU legislation imposes restrictions on the use of both manual and mechanical cervical dislocation based on bird weight (≤3 kg for manual; ≤5 kg for mechanical) and the number of birds killed per day and per stock-worker (70 birds daily for manual), it was essential to document the attitudes of responders regarding those restrictions. The methods used to kill birds has significance for bird welfare and so it is important to document any impacts of those restrictions on the methods used by responders, as almost 70% reported that emergency killing was needed daily. Most participants agreed that limiting manual cervical dislocation by bird weight protects bird welfare and protects against the risk of operator fatigue. Similarly, most respondents agreed that limiting the number of birds that manual cervical dislocation can be performed on daily protects against operator fatigue, despite the only study attempting to investigate this suggested that there was no fatigue risk up to 100 birds for 3 birds types: broilers (2.4 ± 0.9 kg), laying hens (2.8 ± 0.9 kg), and turkeys (12.7 ± 4.0 kg) (Martin et al., 2018b), However, this contrasted with its ability to protect bird welfare, as it could reflect major welfare harms arising from situations where an individual only has access to and is only confident in manual cervical dislocation but due to legal restriction on numbers, must leave birds until the following day if this maximum number was reached. Critically these statements showed agreement in limiting weight thresholds and number of birds per person per day but crucially did not specify figures for these limits. In line with this, most individuals disagreed with the inclusion of a 3 kg weight limit for manual cervical dislocation, a 5 kg limit for mechanical cervical dislocation and the inclusion of a 70-bird maximum per day for manual cervical dislocation. Our results suggest that although operators generally agree that these principles can help, they do not agree with their direct inclusion in legislation and perhaps on the chosen arbitrary and nonscientifically evidenced thresholds. The inclusion of these limits was based on limited data and their inclusion in legislation has been previously contested (Martin et al., 2018b). The direct inclusion of strict weight limits and absolute numbers prevents the flexibility often required for nuanced situations that relies on expert knowledge of poultry stock-workers on the ground to make informed decisions (e.g., a bird with an injured neck or a head/neck deformity may not be suitable for manual or mechanical cervical dislocation). Our findings further challenge the changes made to EU legislation, instead of legal reform we suggest relying on the knowledge of the operators themselves and the development of quality-controlled national training programs and guidance. The direct inclusion of current weight limits poses several potential concerns for bird welfare. It is possible that arbitrary weight limits increase or prolong suffering due to unsuitable kill method selection (e.g., greater pain if the bird is lame and manual cervical dislocation is applied) or delay in kill method application attributable to the requirement of weighing the injured/sick bird and preparation of an alternative method which requires equipment. Overall, we found no major differences in personnel's attitudes towards legislation according to the primary species they worked with. However, we did observe some differences in the way that individuals working with broiler breeders and layer hens agreed with these statements. Individuals working with broiler breeders were more likely to disagree with most of these statements, perhaps given that they were most likely affected by the inclusion of weight limits (most adult birds range from 3-5 kg and are kept for longer production periods; (Aviagen, 2018)). In contrast, individuals working with layer hens were more likely to agree with legislative change. Possible explanations for this could be attributed to the housing systems and health status of hens (Karcher and Mench, 2018), in addition to individuals working with laying hens being relatively unaffected by legislative weight limits, with laying hens in general weighing less than 2 kg. The UK hen population is predominantly free range (63.7% (British Lion Eggs, 2021)), tend to be used and managed for longer periods (up until end of lay 72–85 wk of age (Gerpe et al., 2021)), have lower mortality rates (Schuck-Paim et al., 2021) and may be less accessible for individual bird monitoring due to range and shed furniture compared to broilers, therefore birds may be more likely to be found dead then actively killed by an operator (Bestman and Bikker-Ouwejan, 2020; Schuck-Paim et al., 2021). Therefore, it is possible that these restrictions are unlikely to affect them. In line with this, individuals working with smaller bird species (≤3 kg; broilers and hens) were more likely to disagree that the inclusion of the 3 kg weight limit impacted their method choice, but this was not the case for larger species (>3 kg), where most individuals (65.7%) agreed that the inclusion of the 5 kg weight limit had impacted their choice of method. Therefore, our data suggests that there does not appear to be a clear alternative to manual cervical dislocation, despite stock-worker agreement on killing method property priorities, and it remains the most common method utilized for the individual killing of commercial poultry across the UK.

## CONCLUSIONS

In conclusion, the current study confirms that manual cervical dislocation remains the most common method for killing poultry on farm irrespective of bird species and remains the most preferred method among responders despite legal reform in 2013. The reason behind the popularity of manual cervical dislocation is likely due to the ease and practicality of the method, and due to a lack of alternative methods available across the industry which are suitable and practical for use. This is further hampered by poor knowledge transfer from the scientific literature to relevant stakeholders on what methods perform the best in terms of operation and welfare. This is highlighted by the range of alternative methods available and employed above the 3 kg weight limit for larger birds. Generally, operators’ attitudes towards legislative reform demonstrated the majority largely agree that these principles can protect bird welfare and against operator fatigue, but at present the strict limits set prevent the flexibility often required for nuanced situations. Critically, this survey highlights the importance of providing stakeholders with practical alternatives prior to imposing legislation reforms and the need for proactive knowledge transfer between the scientific community and relevant stakeholders to promote positive change and protect bird welfare.

## DISCLOSURES

The authors declare no conflicts of interest.

## Appendix Supplementary materials

Image, application 1
