# Supplementary material for: A decade on: where is the UK poultry industry for emergency on-farm killing?
Source: Poult Sci. 2023 Feb 21;102(5):102604. doi: 10.1016/j.psj.2023.102604 (PMC10041559; doi:10.1016/j.psj.2023.102604)
Supplement: Supplementary file 1 [file mmc1.pdf]

Supplementary Material S1 for manuscript entitled

“A decade on: Where is the UK poultry industry for emergency on-farm killing?”

Background and informed consent page

<u>Project Title: Assessing attitudes and methods for on-farm despatching of poultry in the United Kingdom</u>

Thank you for your interest to participate in this study. To participate, you must be at least 18 years old, work in the poultry industry and be a resident of the United Kingdom. Participants are asked to complete a short survey which will take **less than 10 minutes**.

Occasionally birds may need to be despatched on-farm for stock-management and to end suffering of sick and injured birds. The aim of this study is to investigate attitudes and preferences of despatching methods for poultry across the UK.

The research is being carried out as part of a Summer Student Scholarship at the University of Edinburgh, Scotland and has been approved by the Royal (Dick) School of Veterinary Studies Human Ethical Review Committee.

The survey has 3 sections to complete:

1) General demographics
2) Identification and preference of despatching methods used
3) Attitudes towards the relevant legislation in the UK.

The demographic questions are all multiple choice and are important for the overall analysis and research outcomes of the study. For sections two and three, there will be a mixture of multiple choice and agreement scales, where you will be asked to select how much you agree or disagree with the statements presented. There are no right or wrong answers.

Your participation in this study is entirely voluntary and you may withdraw at any time before submitting your response. If you choose to withdraw, your responses will not be used.

We do not collect any identifying information such as your name, email or IP address, therefore your responses will be kept anonymous. The data will be stored securely at the University of Edinburgh and will not be shared outside of the research team. By completing this survey, you are consenting for the retention of all anonymous data and the use of the data for future studies to aid animal welfare as well as publication of such studies.

We recognise that the two main topics of discussion, killing animals (i.e., causing death) and animal use in general, can be emotive and potentially distressing subjects to consider. If you find any of the statements presented in this survey distressing, you can stop and withdraw at any point. If you have any questions or concerns about this research, please do not hesitate to contact the researcher, Dr Jessica Martin via jessica.martin@ed.ac.uk. Helpful resources are provided at the end of the survey.

By selecting the 'Yes' option below, you are confirming that:

- You understand the purpose of this study, and that you are able to ask questions about it at any time.
- You understand that you are free to withdraw consent for involvement. However, as the survey is anonymous, once the submit button is clicked, it will not be possible to withdraw.
- You understand that the data collected may – though fully anonymised – appear in publications relevant to this area of research.
- You understand that the data – though fully anonymised – will be available to both the student and the supervisor; and
- By continuing and submitting a response to the survey, you have read and understood the above and have given informed consent.

Yes, I have read and understood the above and give informed consent to participate

No, I do not give consent [Screen out]

Survey

Part 1 – Demographics

1. Which country do you currently reside in?
    a. England
    b. Scotland
    c. Wales
    d. Northern Ireland

2. How would you describe your gender identity?
    a. Male
    b. Female
    c. Other – please specify
    d. Prefer not to say

3. What is your age group?
    a. 18-24 years
    b. 25-34 years
    c. 35-44 years
    d. 45-54 years
    e. 55-64 years
    f. 65 or above

4. What is the highest level of education you have completed?
    a. School level: GCE A level or equivalent
    b. Undergraduate degree
    c. Postgraduate degree
    d. Other – please specify in the text box below

5. Which sector(s) do you currently work in?
    a. Producer
    b. Veterinary services
    c. Breeding
    d. Other

6. Currently, which poultry do you primarily work with?
    a. Broilers
    b. Layer hens
    c. Broiler breeders
    d. Turkeys
    e. Ducks
    f. Geese
    g. Gamebirds

7. Previously, which poultry have you worked with? (please tick all that apply)
    a. Broilers
    b. Layer hens
    c. Turkeys
    d. Ducks
    e. Geese
    f. Gamebirds

8. How many years of experience do you have working with poultry?
    a. 0-1 years
    b. 2-5 years
    c. 6-11 years

d. 12-17 years
e. 18-23 years
f. 24 + years

Part 2 – Identification and preference of despatching methods used

For the following questions, please answer based on which poultry you primarily work with currently (as answered in Q6).

9. Which type of farming system do you work in for the main poultry type you currently work with?
    a. Indoor (non-caged)
    b. Indoor (caged)
    c. Free range
    d. Organic
    e. Mixed/Other

10. Referring to the poultry you currently work with, which stage of production do you primarily work in? (please tick all that apply)
    a. Growing for slaughter/hunting
    b. Egg laying hens
    c. Rearing layer birds
    d. Hatching
    e. Breeding

11. How often do you inspect the poultry houses?
    a. 5+ times a day
    b. 2-4 times a day
    c. Once a day
    d. Every 2-3 days
    e. Once a week

12. Is direct bird care and husbandry your responsibility on a daily basis?
    a. Yes
    b. No

13. In general, how regularly do you have to despatch an individual bird on-farm?
    a. Daily
    b. Couple of times a week
    c. Once a week
    d. Once a month
    e. Couple of times a year
    f. Never

14. Which reason(s) would require an individual bird to be killed on-farm? (please tick all that apply)
    a. Unable to walk
    b. Small/runt bird
    c. Difficulty walking
    d. Respiratory problems
    e. Leg injury
    f. Wing injury
    g. Head injury
    h. Body injury
    i. Loss of feathering
    j. Foot pad dermatitis
    k. Hock burns
    l. Lacking vitality or vigour

m. Central nervous system problems
n. Gastrointestinal problems
o. Deformity
p. other

15. Which on-farm killing method(s) are **available for use** on your site to despatch an individual bird? (please tick all that apply)
    a. Manual cervical dislocation
    b. Mechanical cervical dislocation (Broomstick)
    c. Mechanical cervical dislocation (Pliers)
    d. Mechanical cervical dislocation (Cone)
    e. Mechanical cervical dislocation (Livetec Nex)
    f. Blow to the head
    g. Overdose of anaesthetic (injection)
    h. Captive bolt (cartridge powered)
    i. Captive bolt (non-cartridge powered e.g., compressed air)
    j. Decapitation
    k. Electrical stun-to-kill
    l. Gas
    m. Other

16. Which on-farm killing method(s) are you **confident using** to despatch an individual bird? (please tick all that apply)
    a. Manual cervical dislocation
    b. Mechanical cervical dislocation (Broomstick)
    c. Mechanical cervical dislocation (Pliers)
    d. Mechanical cervical dislocation (Cone)
    e. Mechanical cervical dislocation (Livetec Nex)
    f. Blow to the head
    g. Overdose of anaesthetic (injection)
    h. Captive bolt (cartridge powered)
    i. Captive bolt (non-cartridge powered e.g., compressed air)
    j. Decapitation
    k. Electrical stun-to-kill
    l. Gas
    m. Other

17. How often would you employ each on-farm killing method to despatch an individual bird?
    a. Manual cervical dislocation
    ☐Always ☐ Often ☐ Sometimes ☐ Rarely ☐ Never ☐ Unknown

    b. Mechanical cervical dislocation (Broomstick)
    ☐Always ☐ Often ☐ Sometimes ☐ Rarely ☐ Never ☐ Unknown

    c. Mechanical cervical dislocation (Pliers)
    ☐Always ☐ Often ☐ Sometimes ☐ Rarely ☐ Never ☐ Unknown

    d. Mechanical cervical dislocation (Cone)
    ☐Always ☐ Often ☐ Sometimes ☐ Rarely ☐ Never ☐ Unknown

    e. Mechanical cervical dislocation (Livetec Nex)
    ☐Always ☐ Often ☐ Sometimes ☐ Rarely ☐ Never ☐ Unknown

    f. Blow to the head
    ☐Always ☐ Often ☐ Sometimes ☐ Rarely ☐ Never ☐ Unknown

    g. Overdose of anaesthetic (injection)
    ☐Always ☐ Often ☐ Sometimes ☐ Rarely ☐ Never ☐ Unknown

h. Captive bolt (cartridge powered)
☐Always ☐ Often ☐ Sometimes ☐ Rarely ☐ Never ☐ Unknown

i. Captive bolt (non-cartridge powered e.g., compressed air)
☐Always ☐ Often ☐ Sometimes ☐ Rarely ☐ Never ☐ Unknown

j. Decapitation
☐Always ☐ Often ☐ Sometimes ☐ Rarely ☐ Never ☐ Unknown

k. Electrical stun-to-kill
☐Always ☐ Often ☐ Sometimes ☐ Rarely ☐ Never ☐ Unknown

l. Gas
☐Always ☐ Often ☐ Sometimes ☐ Rarely ☐ Never ☐ Unknown

m. Other
☐Always ☐ Often ☐ Sometimes ☐ Rarely ☐ Never ☐ Unknown

18. Which on-farm killing method is your **personal preferred** method to despatch an individual bird? (please tick only one)
    a. Manual cervical dislocation
    b. Mechanical cervical dislocation (Broomstick)
    c. Mechanical cervical dislocation (Pliers)
    d. Mechanical cervical dislocation (Cone)
    e. Mechanical cervical dislocation (Livetec Nex)
    f. Blow to the head
    g. Overdose of anaesthetic (injection)
    h. Captive bolt (cartridge powered)
    i. Captive bolt (non-cartridge powered e.g., compressed air)
    j. Decapitation
    k. Electrical stun-to-kill
    l. Gas
    m. Other

19. Please rank the following killing method properties based on level of importance (rank 1 being of no importance and rank 10 being most important).
    a. Humane
    b. Quick application
    c. Easy to use
    d. High success rate
    e. Not expensive
    f. Safe for operator
    g. Low maintenance
    h. Low operator fatigue risk
    i. Immediate use in shed
    j. No equipment requirement
    k. Minimal training
    l. Positive public perception

Please indicate to what extent you agree or disagree with each statement for EACH killing method.

20. Manual cervical dislocation:
    a. Humane
    ☐Strongly disagree ☐ Moderately Disagree ☐ Slightly disagree ☐ Slightly Agree ☐ Moderately Agree ☐Strongly Agree ☐Unknown

b. Quick application
☐Strongly disagree ☐ Moderately Disagree ☐ Slightly disagree ☐ Slightly Agree ☐ Moderately Agree ☐Strongly Agree ☐Unknown

c. Easy to use
☐Strongly disagree ☐ Moderately Disagree ☐ Slightly disagree ☐ Slightly Agree ☐ Moderately Agree ☐Strongly Agree ☐Unknown

d. High success rate
☐Strongly disagree ☐ Moderately Disagree ☐ Slightly disagree ☐ Slightly Agree ☐ Moderately Agree ☐Strongly Agree ☐Unknown

e. Not expensive
☐Strongly disagree ☐ Moderately Disagree ☐ Slightly disagree ☐ Slightly Agree ☐ Moderately Agree ☐Strongly Agree ☐Unknown

f. Safe for operator
☐Strongly disagree ☐ Moderately Disagree ☐ Slightly disagree ☐ Slightly Agree ☐ Moderately Agree ☐Strongly Agree ☐Unknown

g. Low maintenance
☐Strongly disagree ☐ Moderately Disagree ☐ Slightly disagree ☐ Slightly Agree ☐ Moderately Agree ☐Strongly Agree ☐Unknown

h. Low operator fatigue risk
☐Strongly disagree ☐ Moderately Disagree ☐ Slightly disagree ☐ Slightly Agree ☐ Moderately Agree ☐Strongly Agree ☐Unknown

i. Minimal training
☐Strongly disagree ☐ Moderately Disagree ☐ Slightly disagree ☐ Slightly Agree ☐ Moderately Agree ☐Strongly Agree ☐Unknown

j. No bird size constraints
☐Strongly disagree ☐ Moderately Disagree ☐ Slightly disagree ☐ Slightly Agree ☐ Moderately Agree ☐Strongly Agree ☐Unknown

k. Positive public perception
☐Strongly disagree ☐ Moderately Disagree ☐ Slightly disagree ☐ Slightly Agree ☐ Moderately Agree ☐Strongly Agree ☐Unknown

21. Mechanical cervical dislocation (general):

a. Humane
☐Strongly disagree ☐ Moderately Disagree ☐ Slightly disagree ☐ Slightly Agree ☐ Moderately Agree ☐Strongly Agree ☐Unknown

b. Quick application
☐Strongly disagree ☐ Moderately Disagree ☐ Slightly disagree ☐ Slightly Agree ☐ Moderately Agree ☐Strongly Agree ☐Unknown

c. Easy to use
☐Strongly disagree ☐ Moderately Disagree ☐ Slightly disagree ☐ Slightly Agree ☐ Moderately Agree ☐Strongly Agree ☐Unknown

d. High success rate
☐Strongly disagree ☐ Moderately Disagree ☐ Slightly disagree ☐ Slightly Agree ☐ Moderately Agree ☐Strongly Agree ☐Unknown

e. Not expensive

☐Strongly disagree ☐ Moderately Disagree ☐ Slightly disagree ☐ Slightly Agree ☐ Moderately Agree ☐Strongly Agree ☐Unknown

f. Safe for operator
☐Strongly disagree ☐ Moderately Disagree ☐ Slightly disagree ☐ Slightly Agree ☐ Moderately Agree ☐Strongly Agree ☐Unknown

g. Low maintenance
☐Strongly disagree ☐ Moderately Disagree ☐ Slightly disagree ☐ Slightly Agree ☐ Moderately Agree ☐Strongly Agree ☐Unknown

h. Low operator fatigue risk
☐Strongly disagree ☐ Moderately Disagree ☐ Slightly disagree ☐ Slightly Agree ☐ Moderately Agree ☐Strongly Agree ☐Unknown

i. Minimal training
☐Strongly disagree ☐ Moderately Disagree ☐ Slightly disagree ☐ Slightly Agree ☐ Moderately Agree ☐Strongly Agree ☐Unknown

j. No bird size constraints
☐Strongly disagree ☐ Moderately Disagree ☐ Slightly disagree ☐ Slightly Agree ☐ Moderately Agree ☐Strongly Agree ☐Unknown

k. Positive public perception
☐Strongly disagree ☐ Moderately Disagree ☐ Slightly disagree ☐ Slightly Agree ☐ Moderately Agree ☐Strongly Agree ☐Unknown

22. Mechanical cervical dislocation (Livetec Nex):

a. Humane
☐Strongly disagree ☐ Moderately Disagree ☐ Slightly disagree ☐ Slightly Agree ☐ Moderately Agree ☐Strongly Agree ☐Unknown

b. Quick application
☐Strongly disagree ☐ Moderately Disagree ☐ Slightly disagree ☐ Slightly Agree ☐ Moderately Agree ☐Strongly Agree ☐Unknown

c. Easy to use
☐Strongly disagree ☐ Moderately Disagree ☐ Slightly disagree ☐ Slightly Agree ☐ Moderately Agree ☐Strongly Agree ☐Unknown

d. High success rate
☐Strongly disagree ☐ Moderately Disagree ☐ Slightly disagree ☐ Slightly Agree ☐ Moderately Agree ☐Strongly Agree ☐Unknown

e. Not expensive
☐Strongly disagree ☐ Moderately Disagree ☐ Slightly disagree ☐ Slightly Agree ☐ Moderately Agree ☐Strongly Agree ☐Unknown

f. Safe for operator
☐Strongly disagree ☐ Moderately Disagree ☐ Slightly disagree ☐ Slightly Agree ☐ Moderately Agree ☐Strongly Agree ☐Unknown

g. Low maintenance
☐Strongly disagree ☐ Moderately Disagree ☐ Slightly disagree ☐ Slightly Agree ☐ Moderately Agree ☐Strongly Agree ☐Unknown

h. Low operator fatigue risk

☐Strongly disagree ☐ Moderately Disagree ☐ Slightly disagree ☐ Slightly Agree ☐ Moderately Agree ☐Strongly Agree ☐Unknown

i. Minimal training
☐Strongly disagree ☐ Moderately Disagree ☐ Slightly disagree ☐ Slightly Agree ☐ Moderately Agree ☐Strongly Agree ☐Unknown

j. No bird size constraints
☐Strongly disagree ☐ Moderately Disagree ☐ Slightly disagree ☐ Slightly Agree ☐ Moderately Agree ☐Strongly Agree ☐Unknown

k. Positive public perception
☐Strongly disagree ☐ Moderately Disagree ☐ Slightly disagree ☐ Slightly Agree ☐ Moderately Agree ☐Strongly Agree ☐Unknown

23. Captive bolt (cartridge powered)

a. Humane
☐Strongly disagree ☐ Moderately Disagree ☐ Slightly disagree ☐ Slightly Agree ☐ Moderately Agree ☐Strongly Agree ☐Unknown

b. Quick application
☐Strongly disagree ☐ Moderately Disagree ☐ Slightly disagree ☐ Slightly Agree ☐ Moderately Agree ☐Strongly Agree ☐Unknown

c. Easy to use
☐Strongly disagree ☐ Moderately Disagree ☐ Slightly disagree ☐ Slightly Agree ☐ Moderately Agree ☐Strongly Agree ☐Unknown

d. High success rate
☐Strongly disagree ☐ Moderately Disagree ☐ Slightly disagree ☐ Slightly Agree ☐ Moderately Agree ☐Strongly Agree ☐Unknown

e. Not expensive
☐Strongly disagree ☐ Moderately Disagree ☐ Slightly disagree ☐ Slightly Agree ☐ Moderately Agree ☐Strongly Agree ☐Unknown

f. Safe for operator
☐Strongly disagree ☐ Moderately Disagree ☐ Slightly disagree ☐ Slightly Agree ☐ Moderately Agree ☐Strongly Agree ☐Unknown

g. Low maintenance
☐Strongly disagree ☐ Moderately Disagree ☐ Slightly disagree ☐ Slightly Agree ☐ Moderately Agree ☐Strongly Agree ☐Unknown

h. Low operator fatigue risk
☐Strongly disagree ☐ Moderately Disagree ☐ Slightly disagree ☐ Slightly Agree ☐ Moderately Agree ☐Strongly Agree ☐Unknown

i. Minimal training
☐Strongly disagree ☐ Moderately Disagree ☐ Slightly disagree ☐ Slightly Agree ☐ Moderately Agree ☐Strongly Agree ☐Unknown

j. No bird size constraints
☐Strongly disagree ☐ Moderately Disagree ☐ Slightly disagree ☐ Slightly Agree ☐ Moderately Agree ☐Strongly Agree ☐Unknown

k. Positive public perception

☐Strongly disagree ☐ Moderately Disagree ☐ Slightly disagree ☐ Slightly Agree ☐ Moderately Agree ☐Strongly Agree ☐Unknown

24. Captive bolt (non-cartridge powered)
    a. Humane
       ☐Strongly disagree ☐ Moderately Disagree ☐ Slightly disagree ☐ Slightly Agree ☐ Moderately Agree ☐Strongly Agree ☐Unknown
    b. Quick application
       ☐Strongly disagree ☐ Moderately Disagree ☐ Slightly disagree ☐ Slightly Agree ☐ Moderately Agree ☐Strongly Agree ☐Unknown
    c. Easy to use
       ☐Strongly disagree ☐ Moderately Disagree ☐ Slightly disagree ☐ Slightly Agree ☐ Moderately Agree ☐Strongly Agree ☐Unknown
    d. High success rate
       ☐Strongly disagree ☐ Moderately Disagree ☐ Slightly disagree ☐ Slightly Agree ☐ Moderately Agree ☐Strongly Agree ☐Unknown
    e. Not expensive
       ☐Strongly disagree ☐ Moderately Disagree ☐ Slightly disagree ☐ Slightly Agree ☐ Moderately Agree ☐Strongly Agree ☐Unknown
    f. Safe for operator
       ☐Strongly disagree ☐ Moderately Disagree ☐ Slightly disagree ☐ Slightly Agree ☐ Moderately Agree ☐Strongly Agree ☐Unknown
    g. Low maintenance
       ☐Strongly disagree ☐ Moderately Disagree ☐ Slightly disagree ☐ Slightly Agree ☐ Moderately Agree ☐Strongly Agree ☐Unknown
    h. Low operator fatigue risk
       ☐Strongly disagree ☐ Moderately Disagree ☐ Slightly disagree ☐ Slightly Agree ☐ Moderately Agree ☐Strongly Agree ☐Unknown
    i. Minimal training
       ☐Strongly disagree ☐ Moderately Disagree ☐ Slightly disagree ☐ Slightly Agree ☐ Moderately Agree ☐Strongly Agree ☐Unknown
    j. No bird size constraints
       ☐Strongly disagree ☐ Moderately Disagree ☐ Slightly disagree ☐ Slightly Agree ☐ Moderately Agree ☐Strongly Agree ☐Unknown
    k. Positive public perception
       ☐Strongly disagree ☐ Moderately Disagree ☐ Slightly disagree ☐ Slightly Agree ☐ Moderately Agree ☐Strongly Agree ☐Unknown

25. Overdose of anaesthetic (injection)
    a. Humane
       ☐Strongly disagree ☐ Moderately Disagree ☐ Slightly disagree ☐ Slightly Agree ☐ Moderately Agree ☐Strongly Agree ☐Unknown
    b. Quick application
       ☐Strongly disagree ☐ Moderately Disagree ☐ Slightly disagree ☐ Slightly Agree ☐ Moderately Agree ☐Strongly Agree ☐Unknown
    c. Easy to use

☐Strongly disagree ☐ Moderately Disagree ☐ Slightly disagree ☐ Slightly Agree ☐ Moderately Agree ☐Strongly Agree ☐Unknown

d. High success rate
☐Strongly disagree ☐ Moderately Disagree ☐ Slightly disagree ☐ Slightly Agree ☐ Moderately Agree ☐Strongly Agree ☐Unknown

e. Not expensive
☐Strongly disagree ☐ Moderately Disagree ☐ Slightly disagree ☐ Slightly Agree ☐ Moderately Agree ☐Strongly Agree ☐Unknown

f. Safe for operator
☐Strongly disagree ☐ Moderately Disagree ☐ Slightly disagree ☐ Slightly Agree ☐ Moderately Agree ☐Strongly Agree ☐Unknown

g. Low maintenance
☐Strongly disagree ☐ Moderately Disagree ☐ Slightly disagree ☐ Slightly Agree ☐ Moderately Agree ☐Strongly Agree ☐Unknown

h. Low operator fatigue risk
☐Strongly disagree ☐ Moderately Disagree ☐ Slightly disagree ☐ Slightly Agree ☐ Moderately Agree ☐Strongly Agree ☐Unknown

i. Minimal training
☐Strongly disagree ☐ Moderately Disagree ☐ Slightly disagree ☐ Slightly Agree ☐ Moderately Agree ☐Strongly Agree ☐Unknown

j. No bird size constraints
☐Strongly disagree ☐ Moderately Disagree ☐ Slightly disagree ☐ Slightly Agree ☐ Moderately Agree ☐Strongly Agree ☐Unknown

k. Positive public perception
☐Strongly disagree ☐ Moderately Disagree ☐ Slightly disagree ☐ Slightly Agree ☐ Moderately Agree ☐Strongly Agree ☐Unknown

26. Blow to the head

a. Humane
☐Strongly disagree ☐ Moderately Disagree ☐ Slightly disagree ☐ Slightly Agree ☐ Moderately Agree ☐Strongly Agree ☐Unknown

b. Quick application
☐Strongly disagree ☐ Moderately Disagree ☐ Slightly disagree ☐ Slightly Agree ☐ Moderately Agree ☐Strongly Agree ☐Unknown

c. Easy to use
☐Strongly disagree ☐ Moderately Disagree ☐ Slightly disagree ☐ Slightly Agree ☐ Moderately Agree ☐Strongly Agree ☐Unknown

d. High success rate
☐Strongly disagree ☐ Moderately Disagree ☐ Slightly disagree ☐ Slightly Agree ☐ Moderately Agree ☐Strongly Agree ☐Unknown

e. Not expensive
☐Strongly disagree ☐ Moderately Disagree ☐ Slightly disagree ☐ Slightly Agree ☐ Moderately Agree ☐Strongly Agree ☐Unknown

f. Safe for operator

☐Strongly disagree ☐ Moderately Disagree ☐ Slightly disagree ☐ Slightly Agree ☐ Moderately Agree ☐Strongly Agree ☐Unknown

g. Low maintenance
☐Strongly disagree ☐ Moderately Disagree ☐ Slightly disagree ☐ Slightly Agree ☐ Moderately Agree ☐Strongly Agree ☐Unknown

h. Low operator fatigue risk
☐Strongly disagree ☐ Moderately Disagree ☐ Slightly disagree ☐ Slightly Agree ☐ Moderately Agree ☐Strongly Agree ☐Unknown

i. Minimal training
☐Strongly disagree ☐ Moderately Disagree ☐ Slightly disagree ☐ Slightly Agree ☐ Moderately Agree ☐Strongly Agree ☐Unknown

j. No bird size constraints
☐Strongly disagree ☐ Moderately Disagree ☐ Slightly disagree ☐ Slightly Agree ☐ Moderately Agree ☐Strongly Agree ☐Unknown

k. Positive public perception
☐Strongly disagree ☐ Moderately Disagree ☐ Slightly disagree ☐ Slightly Agree ☐ Moderately Agree ☐Strongly Agree ☐Unknown

27. Electrical stun-to-kill

a. Humane
☐Strongly disagree ☐ Moderately Disagree ☐ Slightly disagree ☐ Slightly Agree ☐ Moderately Agree ☐Strongly Agree ☐Unknown

b. Quick application
☐Strongly disagree ☐ Moderately Disagree ☐ Slightly disagree ☐ Slightly Agree ☐ Moderately Agree ☐Strongly Agree ☐Unknown

c. Easy to use
☐Strongly disagree ☐ Moderately Disagree ☐ Slightly disagree ☐ Slightly Agree ☐ Moderately Agree ☐Strongly Agree ☐Unknown

d. High success rate
☐Strongly disagree ☐ Moderately Disagree ☐ Slightly disagree ☐ Slightly Agree ☐ Moderately Agree ☐Strongly Agree ☐Unknown

e. Not expensive
☐Strongly disagree ☐ Moderately Disagree ☐ Slightly disagree ☐ Slightly Agree ☐ Moderately Agree ☐Strongly Agree ☐Unknown

f. Safe for operator
☐Strongly disagree ☐ Moderately Disagree ☐ Slightly disagree ☐ Slightly Agree ☐ Moderately Agree ☐Strongly Agree ☐Unknown

g. Low maintenance
☐Strongly disagree ☐ Moderately Disagree ☐ Slightly disagree ☐ Slightly Agree ☐ Moderately Agree ☐Strongly Agree ☐Unknown

h. Low operator fatigue risk
☐Strongly disagree ☐ Moderately Disagree ☐ Slightly disagree ☐ Slightly Agree ☐ Moderately Agree ☐Strongly Agree ☐Unknown

i. Minimal training

☐Strongly disagree ☐ Moderately Disagree ☐ Slightly disagree ☐ Slightly Agree ☐ Moderately Agree ☐Strongly Agree ☐Unknown

j. No bird size constraints
☐Strongly disagree ☐ Moderately Disagree ☐ Slightly disagree ☐ Slightly Agree ☐ Moderately Agree ☐Strongly Agree ☐Unknown

k. Positive public perception
☐Strongly disagree ☐ Moderately Disagree ☐ Slightly disagree ☐ Slightly Agree ☐ Moderately Agree ☐Strongly Agree ☐Unknown

28. Gas

a. Humane
☐Strongly disagree ☐ Moderately Disagree ☐ Slightly disagree ☐ Slightly Agree ☐ Moderately Agree ☐Strongly Agree ☐Unknown

b. Quick application
☐Strongly disagree ☐ Moderately Disagree ☐ Slightly disagree ☐ Slightly Agree ☐ Moderately Agree ☐Strongly Agree ☐Unknown

c. Easy to use
☐Strongly disagree ☐ Moderately Disagree ☐ Slightly disagree ☐ Slightly Agree ☐ Moderately Agree ☐Strongly Agree ☐Unknown

d. High success rate
☐Strongly disagree ☐ Moderately Disagree ☐ Slightly disagree ☐ Slightly Agree ☐ Moderately Agree ☐Strongly Agree ☐Unknown

e. Not expensive
☐Strongly disagree ☐ Moderately Disagree ☐ Slightly disagree ☐ Slightly Agree ☐ Moderately Agree ☐Strongly Agree ☐Unknown

f. Safe for operator
☐Strongly disagree ☐ Moderately Disagree ☐ Slightly disagree ☐ Slightly Agree ☐ Moderately Agree ☐Strongly Agree ☐Unknown

g. Low maintenance
☐Strongly disagree ☐ Moderately Disagree ☐ Slightly disagree ☐ Slightly Agree ☐ Moderately Agree ☐Strongly Agree ☐Unknown

h. Low operator fatigue risk
☐Strongly disagree ☐ Moderately Disagree ☐ Slightly disagree ☐ Slightly Agree ☐ Moderately Agree ☐Strongly Agree ☐Unknown

i. Minimal training
☐Strongly disagree ☐ Moderately Disagree ☐ Slightly disagree ☐ Slightly Agree ☐ Moderately Agree ☐Strongly Agree ☐Unknown

j. No bird size constraints
☐Strongly disagree ☐ Moderately Disagree ☐ Slightly disagree ☐ Slightly Agree ☐ Moderately Agree ☐Strongly Agree ☐Unknown

k. Positive public perception
☐Strongly disagree ☐ Moderately Disagree ☐ Slightly disagree ☐ Slightly Agree ☐ Moderately Agree ☐Strongly Agree ☐Unknown

29. Decapitation
    a. Humane
    ☐Strongly disagree ☐ Moderately Disagree ☐ Slightly disagree ☐ Slightly Agree ☐ Moderately Agree ☐Strongly Agree ☐Unknown

    b. Quick application
    ☐Strongly disagree ☐ Moderately Disagree ☐ Slightly disagree ☐ Slightly Agree ☐ Moderately Agree ☐Strongly Agree ☐Unknown

    c. Easy to use
    ☐Strongly disagree ☐ Moderately Disagree ☐ Slightly disagree ☐ Slightly Agree ☐ Moderately Agree ☐Strongly Agree ☐Unknown

    d. High success rate
    ☐Strongly disagree ☐ Moderately Disagree ☐ Slightly disagree ☐ Slightly Agree ☐ Moderately Agree ☐Strongly Agree ☐Unknown

    e. Not expensive
    ☐Strongly disagree ☐ Moderately Disagree ☐ Slightly disagree ☐ Slightly Agree ☐ Moderately Agree ☐Strongly Agree ☐Unknown

    f. Safe for operator
    ☐Strongly disagree ☐ Moderately Disagree ☐ Slightly disagree ☐ Slightly Agree ☐ Moderately Agree ☐Strongly Agree ☐Unknown

    g. Low maintenance
    ☐Strongly disagree ☐ Moderately Disagree ☐ Slightly disagree ☐ Slightly Agree ☐ Moderately Agree ☐Strongly Agree ☐Unknown

    h. Low operator fatigue risk
    ☐Strongly disagree ☐ Moderately Disagree ☐ Slightly disagree ☐ Slightly Agree ☐ Moderately Agree ☐Strongly Agree ☐Unknown

    i. Minimal training
    ☐Strongly disagree ☐ Moderately Disagree ☐ Slightly disagree ☐ Slightly Agree ☐ Moderately Agree ☐Strongly Agree ☐Unknown

    j. No bird size constraints
    ☐Strongly disagree ☐ Moderately Disagree ☐ Slightly disagree ☐ Slightly Agree ☐ Moderately Agree ☐Strongly Agree ☐Unknown

    k. Positive public perception
    ☐Strongly disagree ☐ Moderately Disagree ☐ Slightly disagree ☐ Slightly Agree ☐ Moderately Agree ☐Strongly Agree ☐Unknown

Part 3 – Attitudes towards the relevant legislation in the UK

In 2013 there was a change to the EU and UK legislation regarding the use of manual and mechanical cervical dislocation for despatching poultry.

Please indicate to what extent you agree or disagree *with each of the following statements:*

1. Limiting manual cervical dislocation by bird weight protects bird welfare.
   ☐Strongly disagree ☐ Moderately Disagree ☐ Slightly disagree ☐ Slightly Agree ☐ Moderately Agree ☐Strongly Agree

2. Restricting the number of birds manual cervical dislocation can be performed on daily protects bird welfare.
☐Strongly disagree ☐ Moderately Disagree ☐ Slightly disagree ☐ Slightly Agree ☐ Moderately Agree ☐Strongly Agree

3. Limiting manual cervical dislocation by bird weight protects against operator fatigue.
☐Strongly disagree ☐ Moderately Disagree ☐ Slightly disagree ☐ Slightly Agree ☐ Moderately Agree ☐Strongly Agree

4. Restricting the number of birds manual cervical dislocation can be performed on daily protects against operator fatigue.
☐Strongly disagree ☐ Moderately Disagree ☐ Slightly disagree ☐ Slightly Agree ☐ Moderately Agree ☐Strongly Agree

5. A weight limit of 3kg for manual cervical dislocation is acceptable.
☐Strongly disagree ☐ Moderately Disagree ☐ Slightly disagree ☐ Slightly Agree ☐ Moderately Agree ☐Strongly Agree

6. The restriction of applying manual cervical dislocation to 70 birds per person per day is acceptable.
☐Strongly disagree ☐ Moderately Disagree ☐ Slightly disagree ☐ Slightly Agree ☐ Moderately Agree ☐Strongly Agree

7. The weight limit of 3kg for manual cervical dislocation impacts despatching method choice.
☐Strongly disagree ☐ Moderately Disagree ☐ Slightly disagree ☐ Slightly Agree ☐ Moderately Agree ☐Strongly Agree

8. The weight limit of 5kg for mechanical cervical dislocation impacts despatching method choice.
☐Strongly disagree ☐ Moderately Disagree ☐ Slightly disagree ☐ Slightly Agree ☐ Moderately Agree ☐Strongly Agree

9. A weight limit of 5kg for mechanical cervical dislocation is acceptable.
☐Strongly disagree ☐ Moderately Disagree ☐ Slightly disagree ☐ Slightly Agree ☐ Moderately Agree ☐Strongly Agree

10. The legislation changes in 2013 impacted my method selection for despatching individual birds on-farm.
☐Strongly disagree ☐ Moderately Disagree ☐ Slightly disagree ☐ Slightly Agree ☐ Moderately Agree ☐Strongly Agree

Thank You Page

You have now completed this survey and your responses have been recorded.

If you have any further questions about this study, please do not hesitate to contact Dr Jessica Martin via jessica.martin@ed.ac.uk.

If you are concerned about the despatching of individual birds on-farm and would like to know more, you can find some information here:

- Humane Slaughter Association

Thank you, your participation in this research is greatly appreciated.
